# Relationships Between Expressions and Variants of the Myosin−Binding Protein C1 Gene and Fatty Acid Composition in Longissimus Thoracis Muscle of Grazing Sonid Sheep

**DOI:** 10.1002/fsn3.71057

**Published:** 2025-10-18

**Authors:** Yilin Wang, Xinyue An, Terigele Li, Takahisa Yamada, Lai Da, Guifang Cao, Ming Cang, Yongli Song, Bin Tong

**Affiliations:** ^1^ The State Key Laboratory of Reproductive Regulation and Breeding of Grassland Livestock, School of Life Sciences Inner Mongolia University Hohhot China; ^2^ Inner Mongolia Agriculture Animal Husbandry Fishery and Biology Experiment Research Centre Inner Mongolia Agricultural University Hohhot China; ^3^ Department of Agrobiology, Faculty of Agriculture Niigata University Niigata Japan; ^4^ Institute of Animal Science, Inner Mongolia Academy of Agricultural and Animal Husbandry Sciences Hohhot China

**Keywords:** fatty acid‐associated marker, longissimus thoracis, MYBPC1 expression, Sonid sheep

## Abstract

This study focused on the associations between the expression levels and variants of the myosin−binding protein C1 (*MYBPC1*) gene and fatty acid composition of the *longissimus thoracis* in grazing Sonid sheep. Fifteen novel polymorphisms were identified in the Sonid sheep breed by direct sequencing and then were genotyped in 286 castrated ram lambs using iPLEX MassARRAY. The association study showed that the g.170969609A>G, g.170969730C>T of linkage disequilibrium−1, the g.171019445C>G, g.171047427G>A, c.3282G>A (1094E), and c.3660C>T (1220S) of linkage disequilibrium−3 of *MYBPC1* have significant effects on the compositions of certain long‐chain FAs, including long‐chain saturated fatty acid, C17:1, n6−polyunsaturated fatty acid, C18:1n9c, and C18:2n6c. In addition, the correlation analysis results showed that the high expression levels of *MYBPC1* were positively correlated with certain saturated fatty acids and negatively correlated with certain unsaturated fatty acids. Thus, the correlation results and associated mutations were expected to be genetic selection markers for the fatty acid compositions and meat quality in Sonid sheep.

## Graphical abstract

This study showed that various FA compositions and classes have significant relationships in the *longissimus thoracis* (LT) muscle of grazing Sonid sheep. The results of association studies indicated that novel mutations of the *MYBPC1* gene were significantly associated with some compositions and classes of FA in the LL of grazing Sonid sheep. Thus, the correlation results and associated mutations are expected to be genetic selection markers for the FA composition and nutrition of grazing Sonid sheep.

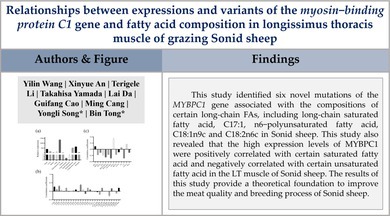

## 1 Introduction

In China, the annual per capita consumption of lamb is rapidly rising. China possesses the largest sheep population in the world, which exceeded 180 million by 2023 (http://www.stats.gov.cn/sj/). As China's economy continues to develop, consumers' demands for the higher quality meat are also steadily increasing. There are approximately 2.206 million Sonid sheep in the Inner Mongolia Autonomous Region (China National Commission of Animal Genetic Resources (CNCAGR) 2011). In view of Sonid sheep's tender and juicy meat, firm muscle layer, high nutritional value, and light odor, more and more consumers have a demand for it (Wang et al. 2021; Yang et al. 2022).

Lamb can provide essential fatty acids (FAs). Also, the proportions of saturated fatty acid (SFA), monounsaturated fatty acid (MUFA), and polyunsaturated fatty acid (PUFA) in meat lamb are of great significance for maintaining normal physiological function in the human body (Tvrzicka et al. 2011; Hoy et al. 2021; Bobiński and Bobińska 2022). The variety and concentration of FAs is influenced by multiple factors and environmental influences (Wood et al. 2008; Ladeira et al. 2018). Among these, the indicators of FA compositions have been found to be moderately to highly heritable (Mortimer et al. 2014; Inoue et al. 2017). Unlike in Australia, where mature and successful extensive finishing systems are used (Ponnampalam, Butler, et al. 2014), Sonid sheep heavily rely on grazing natural grasslands, a practice with a long historical tradition that is expected to continue for a long time. Therefore, understanding the genetic mechanisms that determine meat FA composition is crucial because it may generate new opportunities for improved marker‐assisted selection in breeding, yielding financial gains for the Sonid sheep sector (Pannier et al. 2018). However, compared to other major domestic animals such as beef (Abe et al. 2009; Widmann et al. 2011; Otto et al. 2023) and pork (Muñoz et al. 2013; Estany et al. 2014; Zappaterra et al. 2019), the discovery and exploration of candidate genes affecting FAs in sheep are still limited, and research on candidate genes in Sonid sheep living in desert or semi‐desert Sonid grasslands is even rarer (Guo et al. 2023; Xiang et al. 2025). Therefore, it is necessary to screen and study candidate genes for Sonid sheep breeding.

Myosin−binding protein C1 (*MYBPC1*) is a member of the MYBP−C family, which plays significant roles in the structural and regulatory aspects of muscle functions (Ackermann and Kontrogianni‐Konstantopoulos 2013; James and Robbins 2011). The MYBP‐C family is crucial for providing thick filament stability by interacting, through its C‐terminal region, with titin and the rod segment of sarcomeric myosin (light meromyosin) (Okagaki et al. 1993; Freiburg and Gautel 1996). Furthermore, the MYBPC1 also interacts with muscle‐type creatine kinase and may regulate homeostasis during muscle contraction by coupling with myofibrils (Ha et al. 2013). The *MYBPC1* gene is associated with muscle development and is expressed prenatally (Verardo et al. 2013). It has been demonstrated that the expression levels and single nucleotide polymorphism (SNP) of *MYBPC1* were associated with intramuscular fat (marbling) in Chinese Qinchuan cattle and Japanese Black beef cattle (Tong et al. 2014; Li, Cheng, et al. 2020). The *MYBPC1* gene could be considered a potential candidate gene for enhancing meat quality characteristics in sheep.

This study is designed to accomplish three primary objectives: (1) To identify novel variants within the *MYBPC1* gene in the Sonid sheep breed. (2) To analyze the association between these newly identified *MYBPC1* gene variants and fatty acid composition in Sonid sheep. (3) To evaluate the effects of the expression levels of *MYBPC1* on the FA composition in the *longissimus thoracis* (LT) muscle of sheep. Our study may facilitate effective marker‐assisted selection to promote the meat quality in the Sonid sheep population and provide new insights into the effect of the ovine *MYBPC1* gene on sheep meat quality traits.

## 2 Materials and Methods

### 2.1 Lamb Sample Preparation

This study involved 286 castrated Sonid sheep ram lambs, all approximately 6 months old and born in 2020. These lambs were reared in the Sonid grasslands of Inner Mongolia, utilizing an extensive outdoor grazing system. Initially, they fed on the natural pastures typical of this region until weaning. Post‐weaning, they continued grazing under identical conditions until reaching the age of 6 months, the designated age for slaughter. All lambs included were healthy and represented the offspring of diverse sires and dams, ensuring no recorded parental relationships. Specifically, these lambs were derived from more than 20 unrelated sires, showing the genetic diversity within the study group.

The 286 lambs were slaughtered following the Chinese industry standard (NY/T 1564–2021). Following slaughter, the LT muscle tissue located between the 12^th^ and 13^th^ ribs on the left side of the carcass was immediately harvested. The samples were then swiftly cleared of connective tissue and fat, rapidly frozen using liquid nitrogen, and stored at −80°C for subsequent FA analysis. Out of the 286 lambs, LT muscle samples from 70 randomly selected animals were prepared for RNA extraction of correlation analysis. Furthermore, ten lambs were chosen at random for the *MYBPC1* gene expression profile that included the LT muscle, *semitendinosus* muscle, subcutaneous fat, lung, spleen, large intestine, heart, liver, small intestine, kidney, and stomach.

### 2.2 Determination of Fatty Acid Composition

Intramuscular lipids were extracted from LT samples to produce fatty acid methyl esters (FAME) using a modified Folch method (Folch et al. 1957). For methylation, 200 mg of the extracted sample was treated with both base (0.5 mol/L CH_3_ONa) and acid (14% BF3CH3OH) reagents in separate processes. Each process was conducted in a water bath at 40°C for 20 min (He et al. 2023). Following methylation, the sample was cooled, and FAME was obtained through oscillating extraction with 3 mL of n‐hexane.

FAME analysis was performed in accordance with Dugan and Wood (2018), using gas chromatography (GC) (Varian 450‐GC, Bruker Daltonics Inc., Fremont, USA) equipped with a 100 m × 0.2 μm × 0.25 mm capillary column (SP‐2560, Sigma‐Aldrich, Shanghai, China). The injection port and detector were initially set to 260°C. The column temperature was maintained at 120°C for 5 min, then increased to 230°C at a rate of 3°C/min and held for 3 min, followed by an increase to 240°C at a rate of 1.5°C/min and held for an additional 5 min. Helium was used as the carrier gas at a flow rate of 1 mL/min, with a split ratio of 20:1. Quantification of FAMEs was based on chromatographic peak area calculations. Specific fatty acid peaks were identified by comparing GC retention times to known standards (Supelco 37 Component FAME mix, Sigma‐Aldrich, Shanghai, China) and conjugated linoleic acid (CLA, Shanghai ANPEL Scientific Instrument, Shanghai, China). Each measurement was performed in triplicate.

### 2.3 RT‐qPCR

Using the RNAiso Plus reagent kit, total RNA was isolated from the LT muscle tissue of Sonid sheep (Takara Bio Inc., Dalian, China) and subsequently converted to cDNA with the PrimeScript RT Reagent Kit with gDNA Eraser (Takara Bio Inc., Dalian, China). Using the primers listed in Table 1 and the SYBR Green master mix, RT‐qPCR analysis was performed, employing a system from Bio‐Rad (Bio‐Rad, Hercules, CA, USA). *GAPDH* was employed as an internal control for normalization purposes in this study (Guo et al. 2023). The $2^{-∆∆C_{t}}$ method was used to calculate the relative expression level of the *MYBPC1* gene, normalized to *GAPDH*.

**TABLE 1:** The information of *GAPDH* and *MYBPC1* for RT‐qPCR.

Gene	Primer (5′‐3′)	Size (bp)	Tm (°C)	Accession number
*GAPDH*	F: AATACTGAGATGTCCTTC	140	53.8	NM_001190390.1
	R: TTTATGGTGGTTGATTTC			
*MYBPC1*	F: ATCAAAGCCAAAGAGAACTACGC	184	59.2	XM_012174908.3
	R: TAAAGTCAAGTTCCCCTGCATCG			

Abbreviations: F, Forward primer; R, Reverse primer.

### 2.4 Mutation Identification in *MYBPC1*

For the extraction of genomic DNA from LT muscle tissues, the Wizard Genomic DNA Purification Kit (Promega Corp, Madison, WI, USA) was utilized. The extracted DNA's quality and quantity were evaluated using a NanoDrop spectrophotometer (Thermo Fisher Scientific, Waltham, MA, USA), and its integrity was further confirmed by agarose gel electrophoresis. Only genomic DNA samples displaying an absorbance ratio (OD260 nm/OD280 nm) of ≥ 1.80 and showing no signs of RNA or protein contamination were selected for further analysis. Genomic DNA from ten randomly selected Sonid sheep was submitted to Beijing Novogene Company for 10 × genomic resequencing.

### 2.5 Polymorphism Genotyping Using iPLEX MassARRAY

The MassARRAY SNP genotyping system (Biosciences, San Diego, CA, USA) was employed to analyze the genotypes of 15 mutations within the *MYBPC1* gene across a cohort of 286 Sonid sheep. The Assay Design Suite software (available at http://agenabio.com/assay‐design‐suite2‐0‐software) was utilized, following standard parameters, to generate PCR and extension primers specifically targeting the 15 mutations. These primers were crafted based on sequences surrounding the mutations, extending approximately 100 bases both upstream and downstream (Table 2). The Sequenom MassARRAY iPLEX platform was the tool of choice for determining the genotypes of the individual alleles (Gabriel et al. 2009). The data analysis was conducted with the aid of MassARRAY Typer 4.0 analyzer software (Biosciences, San Diego, CA, USA), ensuring precise interpretation of the genotyping results (Gabriel et al. 2009).

**TABLE 2:** MassARRAY information for the identified variants in the *MYBPC1* gene.

Mutation	Primer (5′‐3′)	Tm (°C)
g.170969337C>T	F: ACGTTGGATGTTGGGTTGGCCCAAAAGTTC	43
	R: ACGTTGGATGTGCCATGGATGTGGACAAAG	
	E: GGTTTGTGGAAAAACCC	
g.170969609A>G	F: ACGTTGGATGGCTCTGGAATTAGGTAGTGG	46.5
	R: ACGTTGGATGTGACTTTTCTTGCCCATGCC	
	E: GGACTTAGGTAGTGGTAATGGATG	
g.170969682G>A	F: ACGTTGGATGTAGCGAAAGATGCCAGACAC	42.9
	R: ACGTTGGATGCAGAGCATTTTCATCATAACC	
	E: AGGTGGGATTAGGAG	
g.170969730C>T	F: ACGTTGGATGCAGAGCATTTTCATCATAACC	45.3
	R: ACGTTGGATGTAGCGAAAGATGCCAGACAC	
	E: CATGCAACCACTAATTGAGTT	
g.170969787C>T	F: ACGTTGGATGCAGAGCATTTTCATCATAACC	36.5
	R: ACGTTGGATGTAGCGAAAGATGCCAGACAC	
	E: TATTAATGGAATCATACAATA	
g.171019445C>G	F: ACGTTGGATGCAATCAGGAATCTGAGAGGG	57.9
	R: ACGTTGGATGCCTGTGTTACTGGGAATTTC	
	E: CTATATGCAGCGGATGGAGAAAGGG	
c.2589G>T (863 V)	F: ACGTTGGATGTGGGCTGAGAGTAGTACTTG	50.1
	R: ACGTTGGATGGTCTGCCAACGGATTCAAGG	
	E: CCTTCACAGCCTTCACACG	
g.171047427G>A	F: ACGTTGGATGAGTATATGCCAGGTACACTC	47.1
	R: ACGTTGGATGGAGGCTCTGAGATAGAGTAG	
	E: GGAGTCCAGGTACACTCATGTAAG	
g.171057982G>A	F: ACGTTGGATGTGACAAAAGAGAGTGCCGTG	58.4
	R: ACGTTGGATGTGTCTTCTCTGTCTCATCAC	
	E: TTGTAGAGGGCTGGACATCCGTGA	
g.171058187C>T	F: ACGTTGGATGCTTAAGGGAAACTGTACTATG	52.1
	R: ACGTTGGATGGCGACTTTTTCCTATCCCTG	
	E: CCCGCATTCATTCCAACATCTGGACAG	
c.3282G>A (1094E)	F: ACGTTGGATGATAGGTGCCTCTGTGAAATC	45
	R: ACGTTGGATGTGTAGTCAACTACGCCAAGG	
	E: ACTCTGTGAAATCGAAGTC	
c.3345A>G (1115A)	F: ACGTTGGATGGCACCTATGTTTACTCAGCC	53.4
	R: ACGTTGGATGTTCCTCTCACACTGCAGTTC	
	E: CATGGGTCAACACCTATGCTGTAGC	
g.171061056A>C	F: ACGTTGGATGTGGATCTTATCCCCCCCTTC	46.3
	R: ACGTTGGATGATCCCAGATCTCGTGTAGAC	
	E: GACCCCCTTCCTAACCT	
c.3660C>T (1220S)	F: ACGTTGGATGTGCCCAGGCCCTTAGTATTC	44.4
	R: ACGTTGGATGCGTGCCTCCTAACATAATTG	
	E: GGGATTAGTATTCCTCAGGGTT	
g.171066159C>G	F: ACGTTGGATGCCACAACCCTACTTAATTCC	45.8
	R: ACGTTGGATGCCCTTCTGTTGAGAAAACTTG	
	E: CCCGACTTAATTCCAGTGGATCT	

Abbreviations: E, Extended primer sequence; F, Forward primer sequence; R, Reverse primer sequence.

### 2.6 Bioinformatics Analysis

Alignment of the sequences of the *MYBPC1* gene across different species was conducted using Clustal Omega, with sequences sourced from UniProt (http://www.uniprot.org). The mRNA secondary structure of the *MYBPC1* gene in sheep was examined employing RNAfold (http://rna.tbi.univie.ac.at/cgi‐bin/RNAWebSuite/RNAfold.cgi). Lastly, transcription factors (TF) binding to the g.170969609A>G SNP were predicted using Jaspar (http://jaspar.genereg.net/), with the relative profile score threshold set at 80%. The heat map was generated by https://www.omicstudio.cn/tool.

### 2.7 Statistical Analysis

Population genetic metrics, including polymorphism information content (PIC), number of effective alleles (n_e_), observed heterozygosity (Ho), and expected heterozygosity (He), were calculated using the methodologies developed by Nei and Roychoudhury (1974). The chi‐squared test was employed to assess allele frequencies of each mutation independently. Linkage disequilibrium (LD) analyses were performed with HaploView 4.2 software, evaluating D′ and *r* ^2^ values to determine linkage strength (Barrett et al. 2005). The association analysis between fatty acid (FA) composition/class and the genotypes of each variant was explored using SPSS 27.0 software (SPSS, Chicago, IL, USA), following the approach described by Guo et al. (2023). A statistical linear model, Y_i_ = *μ* + G_i_ + e_i_, was utilized for analysis, where Y_i_ represents the observed value for each trait, *μ* is the trait's average measurement value, G_i_ indicates the fixed effect of the genotype on the trait, and e_i_ is the standard error associated with the measurement. When the number of sheep with a given genotype was less than ten, their associations and effects could not be reliably estimated. Therefore, animals with this genotype were excluded from the analysis. Using the Bonferroni correction method, the *p*‐values were adjusted for multiple comparisons (Gao et al. 2022). Correlation analyses to explore the relationships between pairs of FA compositions and classes, as well as between the expression levels of *MYBPC1* and each FA composition and class, were conducted using SPSS 27.0. Results were reported as Pearson correlation coefficients.

## 3 Results

### 3.1 Fatty Acid Profiles of the Longissimus Thoracis Muscle

**FIGURE 1:**
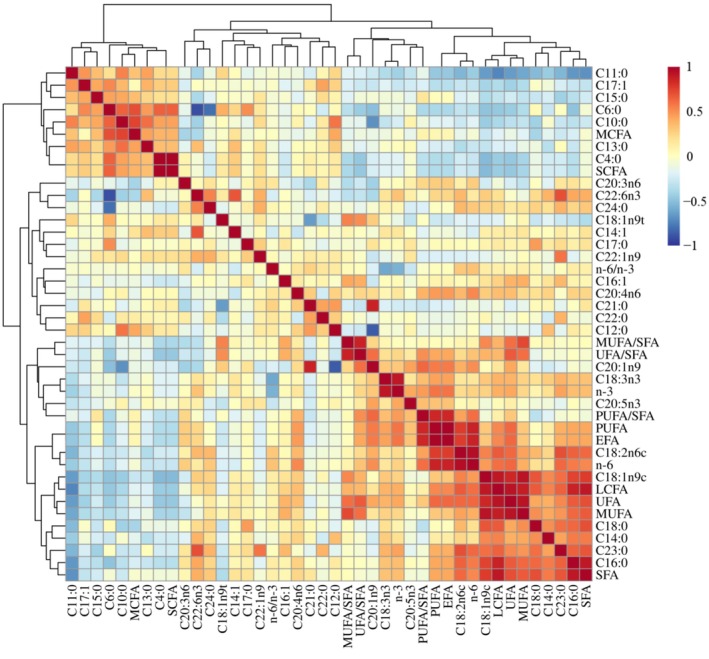
A correlogram depicting the correlation analyses between pairs of traits within fatty acid compositions and classes. Shades of red indicate positive correlations, whereas shades of blue represent negative correlations. The color intensity is proportional to the magnitude of the correlation coefficient. The heat map was generated by https://www.omicstudio.cn/tool.

The 286 LT muscles of Sonid sheep were analyzed for their FA composition and FA classes, which are summarized in Table S1. The table lists 15 types of SFA, seven types of MUFA, and six types of PUFA present in the LT muscle. Figure 1 illustrates the correlation analyses between various traits within FA compositions/classes, offering a visual depiction of their relationships, while the correlation coefficients *r* and their respective *p*‐values are presented in Table S2.

The SFA content of LT muscle was strongly and positively correlated with C16:0, C18:0, C18:1n9c, UFA, and LCFA, with *r* values ranging from 0.704 to 0.912 and *p* < 0.01, and it was strongly and negatively correlated with C10:0, C11:0, and C15:0 with *r* values ranging from −0.689 to −0.246 and *p* < 0.01 (Figure 1, Table S2). The MUFA content of LT muscle was strongly and positively correlated with C18:1n9c, UFA, MUFA/SFA, and LCFA, with *r* values ranging from 0.729 to 0.927 and *p* < 0.01; it was strongly and negatively correlated with C4:0, C10:0, C11:0, and C15:0, with *r* values ranging from −0.601 to −0.308 and *p* < 0.01 (Figure 1, Table S2). The PUFA content of LT muscle was strongly and positively correlated with C18:2n6c, PUFA/SFA, n‐6, and EFA with *r* values ranging from 0.766 to 1.000 and *p* < 0.01, and it was strongly and negatively correlated with C4:0, C6:0, C11:0, C17:1, and C18:1n9t with *r* values ranging from −0.469 to −0.256 and *p* < 0.01 (Figure 1, Table S2).

### 3.2 *MYBPC1* Gene Expression Profiles in Sheep

**FIGURE 2:**
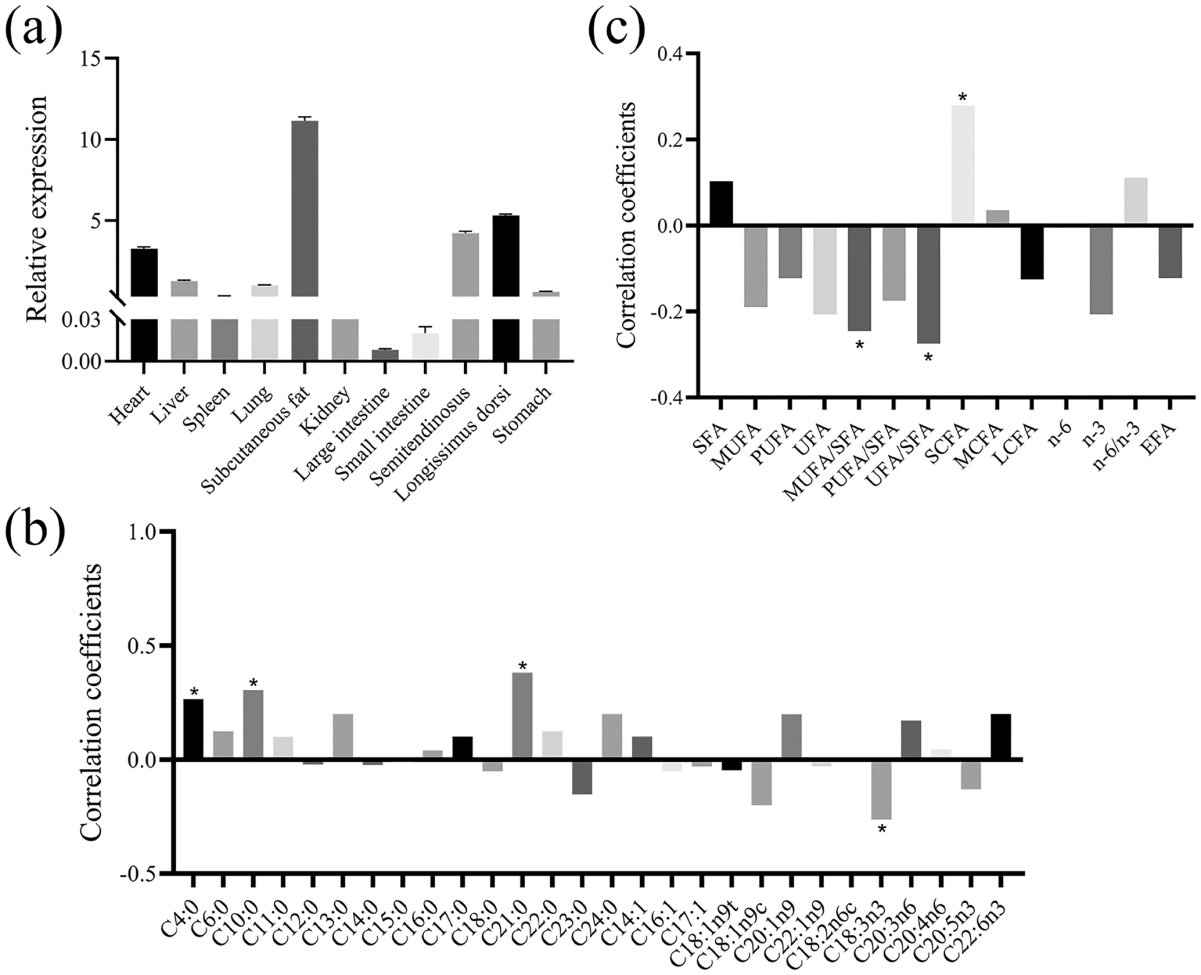
The expression profile of the *MYBPC1* gene in sheep. (a) Relative expression levels of *MYBPC1* genes in the 11 different tissues of Sonid sheep (*n* = 10). (b) Correlations between fatty acid composition and expression levels of *MYBPC1* in the *longissimus thoracis* muscle of 70 Sonid sheep. (c) Correlations between fatty acid classes and expression levels of *MYBPC1* in the *longissimus thoracis* muscle of 70 Sonid sheep. *p < 0.05.

Figure 2a displays the gene expression profiles of the *MYBPC1* gene across 11 different tissues in Sonid sheep. The expression levels of *MYBPC1* were relatively high in subcutaneous fat, heart tissue, *semitendinosus* tissue, and LT (Figure 2a). The expression levels of the *MYBPC1* gene showed a positive correlation with C4:0, C10:0, heneicosylic acid (C21:0), and short‐chain fatty acids (SCFA) (*p* < 0.05). The *MYBPC1* gene expressions were negatively correlated with C18:3n3, the ratio of MUFA/SFA, and the ratio of UFA/SFA, respectively (*p* < 0.05; Figure 2b,c).

### 3.3 Variant Discovery in Ovine *MYBPC1*

**FIGURE 3:**
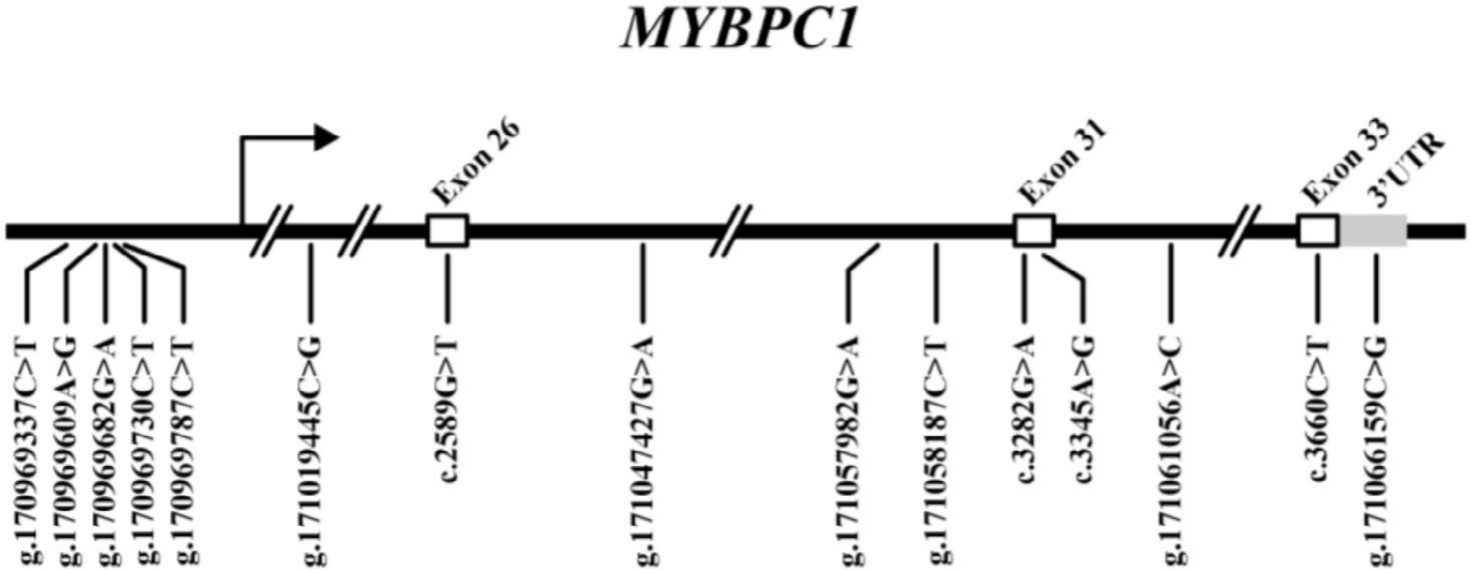
The physical locations of the 15 novel variants of the *MYBPC1* gene in Sonid sheep. The variant sites are located on chromosome 3 in ARS–UI_Ramb_v2.0 (GenBank accession: XM_012174908.3).

The sequence analysis identified 15 novel variants in the *MYBPC1* gene of Sonid sheep. Among them, the five polymorphisms including the g.170969337C>T, g.170969609A>G, g.170969682G>A, g.170969730C>T, and g.170969787C>T in the promoter region of *MYBPC1*, and the g.171019445C>G, g.171047427G>A, g.171057982G>A, g.171058187C>T, and g.171061056A>C in the introns of *MYBPC1*, and the silent c.2589G>T (863 Valerine), c.3282G>A (1094 Glutamic), c.3345A>G (1115 Alanine), and c.3660C>T (1220 Serine) in the exons 26, 31, and 33 of the *MYBPC1* gene, respectively. A single mutation (g.171066159C>G) was identified in the 3′ untranslated region (UTR) of the *MYBPC1* gene (Figure 3).

**FIGURE 4:**
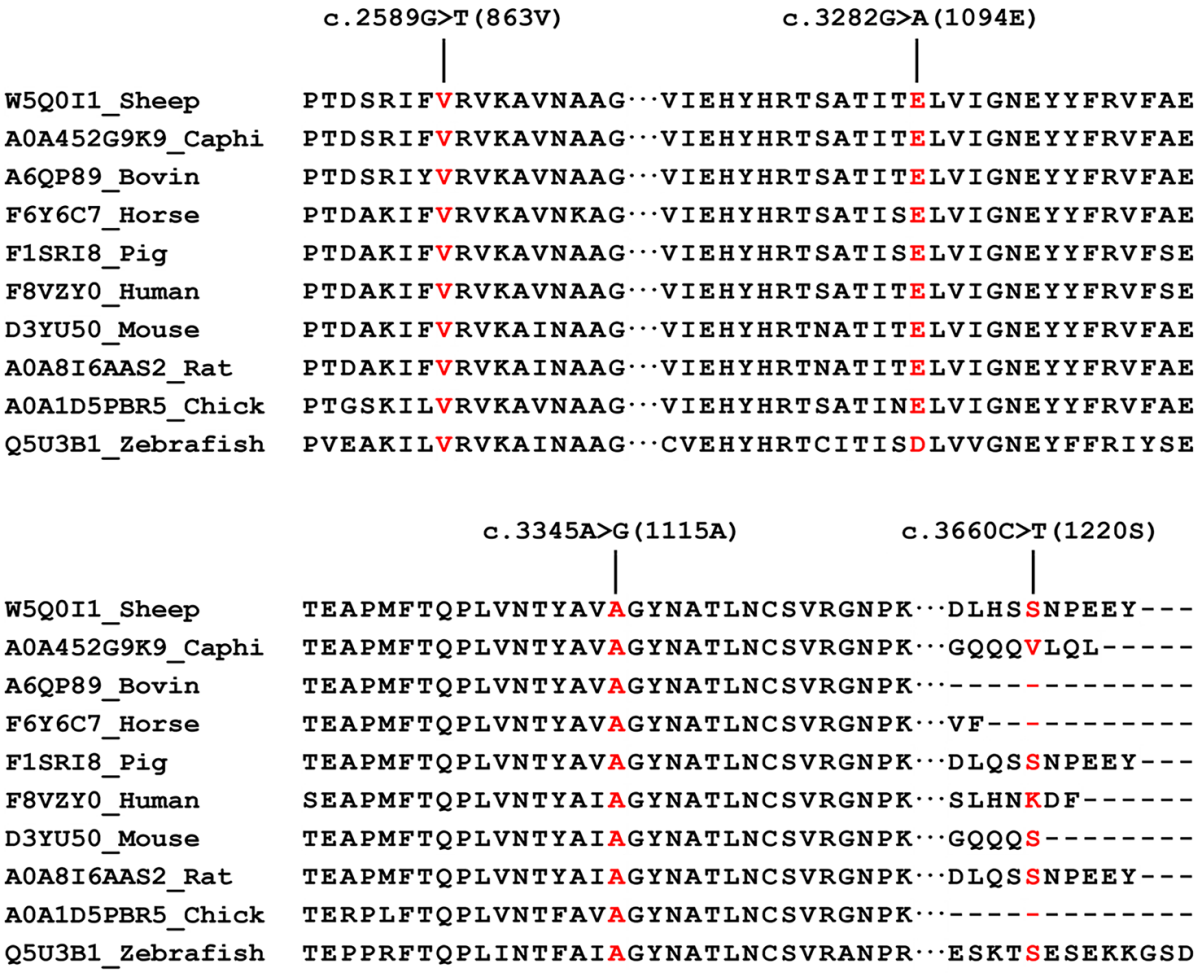
The wild‐type amino acid sequences of MYBPC1 from ten species were aligned, with a focus on the multispecies alignment of the MYBPC1 mutation regions. The amino acid sequences of MYBPC1 for each species were obtained from the Uniprot database.

The c.2589G>T (863 V), c.3282G>A (1094E), c.3345A>G (1115A), and c.3660C>T (1220S) mutations in the exons 26, 31, and 33 of *MYBPC1* caused silent mutations at the 863 V, 1094E, 1115A, and 1220S positions in the amino acid sequence of the MYBPC1 protein, respectively (Figure 4).

For 15 mutations identified in this study, the genotypical and allelic frequencies in the Sonid sheep population, along with the genetic indices, are listed in Table S3, as are the genetic indices (H_o_, H_e_, n_e_, PIC, and Hardy–Weinberg equilibrium). None of the tests conducted on each variant in Sonid sheep detected significant departures at the 5% level. The values of the PIC of the seven variants (g.170969337C>T, g.170969682G>A, g.170969730C>T, g.170969787C>T, g.171058187C>T, c.3345A>G, and g.171061056A>C) presented related low polymorphism in Sonid sheep. The values of the PIC of the eight variants (g.170969609A>G, g.171019445C>G, c.2589G>T, g.171047427G>A, g.171057982G>A, c.3282G>A, c.3660C>T, and g.171066159C>G) demonstrated moderate polymorphism observed in the Sonid sheep population (Table S3).

### 3.4 Linkage Disequilibrium Analysis of Novel Variants in *MYBPC1*

**FIGURE 5:**
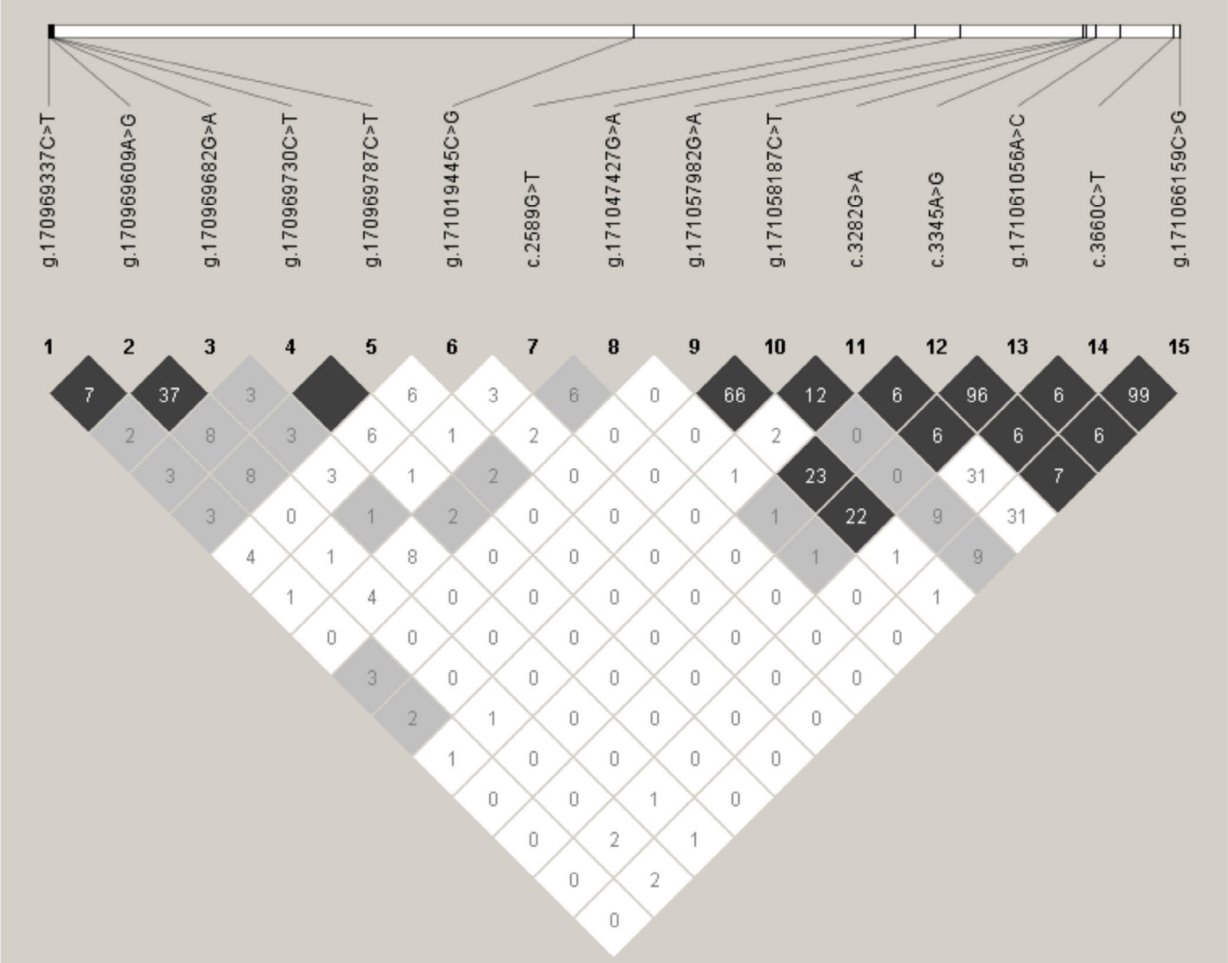
Linkage disequilibrium estimated among *MYBPC1* variations in Sonid sheep. 1: G.170969337C>T, 2: G.170969609A>G, 3: G.170969682G>A, 4: G.170969730C>T, 5: G.170969787C>T, 6: G.171019445C>G, 7: C.2589G>T, 8: G.171047427G>A, 9: G.171057982G>A, 10: G.171058187C>T, 11: C.3282G>A, 12: C.3345A>G, 13: G.171061056A>C, 14: C.3660C>T, 15: G.171066159C>G.

To determine the linkage relationships among 15 variants, we estimated D′ and *r* ^2^ for the experimental Sonid sheep population. The resulting *r* ^2^ indicated that the g.170969730C>T and g.170969787C>T variants were in complete LD in the experimental Sonid sheep by *r* ^2^ = 1.000 (Figure 5). The c.3345A>G and g.171061056A>C variants exhibited nearly complete LD in the experimental Sonid sheep by *r* ^2^ = 0.966 (Figure 5). In the experimental Sonid sheep, the c.3660C>T and g.171066159C>G variants exhibited nearly complete LD by *r* ^2^ = 0.993 (Figure 5). Thus, these LD groups were collectively analyzed and designated as the single locus, denoted as LD‐1, LD‐2, and LD‐3, respectively. Table S4 presents the D′ and *r* ^2^ of the experimental Sonid sheep population.

### 3.5 Associations Between Variants in *MYBPC1* and Fatty Acid

Table 3 presents the results of significant associations between the 15 novel variants of *MYBPC1* and FA compositions/classes in the LT of Sonid sheep, with full results provided in Table S5. At the g.170969609A>G site, the content of C21:0 (*p* < 0.01) of individuals with the AA genotype was significantly more than that of those with the AG genotype (*p* < 0.01; Table 3). For the g.170969730C>T of LD‐1, the contents of C18:0 (*p* < 0.01) of individuals with the CC genotype exhibited significantly higher levels compared to those with the CT genotype (*p* < 0.01; Table 3). At the g.171019445C>G site, the individuals carrying the CC genotype exhibited significantly elevated levels of C15:0 in comparison to those with the CG genotype (*p* < 0.05), and the individuals carrying the CC genotype displayed notably higher levels of C21:0 compared to those with either the CG or GG genotypes (*p* < 0.05; Table 3). For the g.171047427G>A and c.3282G>A mutations, the individuals with the GG genotype had notably lower levels of C21:0 compared to those with the GA genotype, respectively (*p* < 0.01; Table 3 (continued)). For the c.3282G>A mutation, the individuals with the GG genotype had markedly higher levels of n6‐PUFA compared to those with the AA genotype (*p* < 0.05), and the individuals with the GG genotype exhibited notably lower levels of C17:1 compared to those with other genotypes (*p* < 0.05; Table 3 (continued)). In addition, the individuals with the CC genotype of the c.3660C>T site of LD‐3 had significantly higher C17:1 levels when compared to the individuals with the TT genotype (*p* < 0.05), and the C16:0 content and SFA levels in individuals with the CC genotype were significantly lower compared to those with the CT genotype (*p* < 0.05); the individuals carrying the CT genotype showed notably elevated levels of C21:0 compared to those with the TT genotype (*p* < 0.05); the individuals with the CC genotype exhibited notably lower levels of C18:1n9c (*p* < 0.05), C18:2n6c (*p* < 0.05), and long‐chain fatty acids (LCFA) (*p* < 0.05) levels when compared to sheep with the CT and TT genotypes, respectively (*p* < 0.05); the individuals with the CC genotype had significantly lower UFA (*p* < 0.05) and n6‐PUFA (*p* < 0.05) levels when compared to the individuals with the CT and TT genotypes in Sonid sheep (Table 3).

**TABLE 3:** Associations between the variants in *MYBPC1* and fatty acid of the *longissimus thoracis* muscle in Sonid sheep.

Fatty acid	g.170969609A>G			g.170969730C>T in LD‐1		g.171019445C>G		
	Genotype			Genotype		Genotype		
	AA (140)	AG (124)	GG (22)	CC (197)	CT (83)	CC (100)	CG (144)	GG (42)
C15:0	0.77 ± 0.04	0.83 ± 0.05	0.74 ± 0.06	0.81 ± 0.03	0.76 ± 0.05	0.91 ± 0.05^a^	0.73 ± 0.03^a^	0.75 ± 0.07^a^
C16:0	16.96 ± 0.31	16.97 ± 0.31	17.74 ± 0.67	17.25 ± 0.23	16.32 ± 0.45	16.62 ± 0.34	17.14 ± 0.29	17.58 ± 0.63
C18:0	9.42 ± 0.17	9.35 ± 0.18	10.40 ± 0.39	9.78 ± 0.13^b^	8.76 ± 0.23^b^	9.21 ± 0.19	9.66 ± 0.17	9.46 ± 0.31
C21:0	0.76 ± 0.18^b^	0.49 ± 0.04^b^	0.63 ± 0.05^b^	0.58 ± 0.04	0.73 ± 0.26	0.75 ± 0.24^a^	0.59 ± 0.04	0.51 ± 0.06^a^
SFA	30.88 ± 0.49	31.13 ± 0.48	31.98 ± 0.97	31.47 ± 0.37	30.03 ± 0.69	30.58 ± 0.51	31.21 ± 0.47	31.79 ± 0.93
C17:1	0.76 ± 0.03	0.69 ± 0.03	0.65 ± 0.08	0.74 ± 0.02	0.70 ± 0.04	0.75 ± 0.04	0.70 ± 0.03	0.75 ± 0.06
C18:1n9c	15.52 ± 0.40	15.96 ± 0.35	17.40 ± 0.85	16.41 ± 0.30	14.62 ± 0.51	15.43 ± 0.39	16.21 ± 0.40	15.64 ± 0.64
C18:2n6c	4.53 ± 0.11	4.50 ± 0.11	4.66 ± 0.23	4.53 ± 0.09	4.49 ± 0.15	4.42 ± 0.11	4.52 ± 0.10	4.80 ± 0.25
UFA	23.89 ± 0.54	23.86 ± 0.50	25.44 ± 1.23	24.42 ± 0.42	23.07 ± 0.70	23.82 ± 0.53	24.17 ± 0.54	23.82 ± 0.91
LCFA	53.34 ± 0.97	53.48 ± 0.89	56.50 ± 2.14	54.61 ± 0.73	51.33 ± 1.28	52.82 ± 0.97	54.06 ± 0.93	54.16 ± 1.73
n‐6	4.85 ± 0.13	4.68 ± 0.15	5.15 ± 0.28	4.78 ± 0.11	4.80 ± 0.18	4.76 ± 0.14	4.78 ± 0.13	4.94 ± 0.30

Fatty acid	g.171047427G>A			c.3282G>A			c.3660C>T in LD‐3		
	Genotype			Genotype			Genotype		
	GG (173)	GA (101)	AA (12)	GG (67)	GA (133)	AA (86)	CC (62)	CT (135)	TT (89)
C15:0	0.81 ± 0.04	0.78 ± 0.04	0.71 ± 0.12	0.78 ± 0.06	0.81 ± 0.04	0.78 ± 0.05	0.79 ± 0.05	0.82 ± 0.04	0.75 ± 0.05
C16:0	16.80 ± 0.27	17.26 ± 0.36	18.13 ± 1.03	17.39 ± 0.37	16.97 ± 0.31	16.82 ± 0.41	15.96 ± 0.50^a^	17.33 ± 0.28^a^	17.29 ± 0.38^a^
C18:0	9.46 ± 0.14	9.39 ± 0.21	10.23 ± 0.56	9.57 ± 0.24	9.43 ± 0.18	9.46 ± 0.20	9.15 ± 0.26	9.64 ± 0.17	9.43 ± 0.21
C21:0	0.55 ± 0.04^b^	0.76 ± 0.21^b^	0.54 ± 0.10^b^	0.51 ± 0.06^b^	0.75 ± 0.19^b^	0.58 ± 0.05^b^	0.60 ± 0.05^a^	0.71 ± 0.19^a^	0.55 ± 0.07^a^
SFA	30.82 ± 0.42	31.42 ± 0.55	31.80 ± 1.45	31.63 ± 0.61	31.12 ± 0.49	30.57 ± 0.61	29.26 ± 0.79^a^	31.66 ± 0.41^a^	31.46 ± 0.61^a^
C17:1	0.73 ± 0.03	0.70 ± 0.03	0.76 ± 0.09	0.62 ± 0.04^a^	0.77 ± 0.03^a^	0.73 ± 0.03^a^	0.81 ± 0.05^a^	0.74 ± 0.03^a^	0.64 ± 0.04^a^
C18:1n9c	15.68 ± 0.34	15.90 ± 0.43	17.94 ± 1.25	16.79 ± 0.47	15.53 ± 0.39	15.63 ± 0.49	14.32 ± 0.63^a^	16.23 ± 0.35^a^	16.36 ± 0.45^a^
C18:2n6c	4.52 ± 0.10	4.52 ± 0.12	4.65 ± 0.36	4.70 ± 0.14	4.54 ± 0.12	4.36 ± 0.13	4.11 ± 0.16^a^	4.59 ± 0.09^a^	4.72 ± 0.15^a^
UFA	23.82 ± 0.45	24.16 ± 0.58	25.18 ± 2.09	25.33 ± 0.65	23.64 ± 0.52	23.50 ± 0.69	22.22 ± 0.86^a^	24.13 ± 0.45^a^	25.03 ± 0.66^a^
LCFA	53.25 ± 0.81	54.00 ± 1.07	56.22 ± 3.36	55.46 ± 1.14	53.24 ± 0.95	52.84 ± 1.20	50.15 ± 1.52^a^	54.36 ± 0.79^a^	54.98 ± 1.21^a^
n−6	4.75 ± 0.12	4.87 ± 0.14	4.83 ± 0.43	5.11 ± 0.17^a^	4.82 ± 0.14^a^	4.51 ± 0.17^a^	4.37 ± 0.20^a^	4.81 ± 0.12^a^	5.06 ± 0.18^a^

*Note:* Values represent the mean ± standard error.

^a^ Means that the difference between different superscript values within the same line is statistically significant (*p <* 0.05).

^b^ Means that the difference between different superscript values within the same line is statistically significant (*p <* 0.01).

### 3.6 The Secondary Structure of the *MYBPC1* mRNA

**FIGURE 6:**
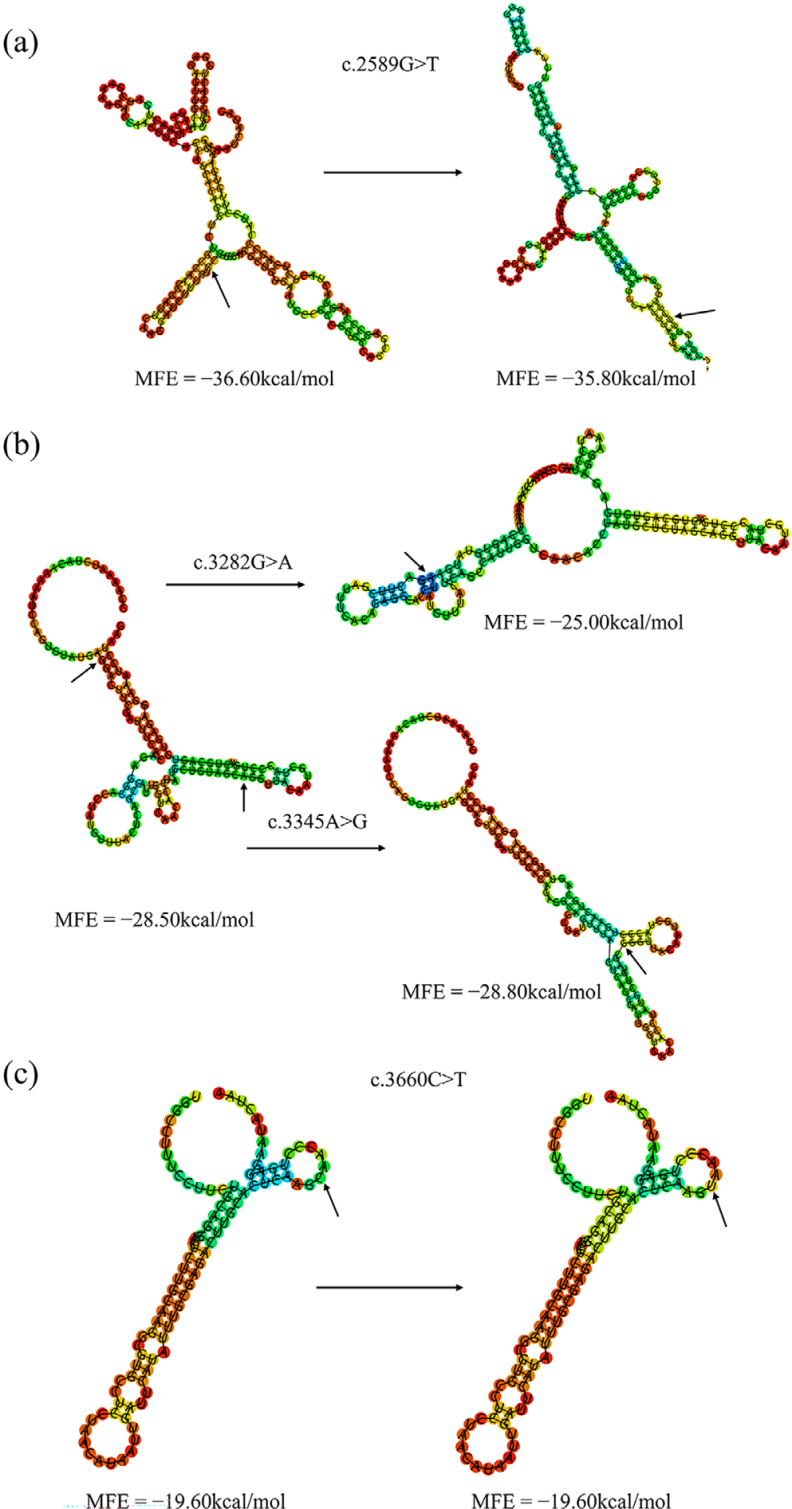
The minimum free energy (MFE) of secondary structure in exons 26, 31, and 33 of the *MYBPC1* gene. The MFE prediction in terms of the secondary structure and free energy: (a) The secondary structure of the wild‐type mRNA for exon 26 and the alterations in secondary structure caused by the c.2589G>T mutation; (b) The secondary structure of the wild‐type mRNA for exon 31 and the changes in secondary structure caused by the c.3282G>A and c.3345A>G mutations; (c) The secondary structure of the wild‐type mRNA for exon 33 and the changes in secondary structure caused by the c.3660C>T mutation. The structure is colored based on base‐pairing probabilities.

The minimum free energy (MFE) values for the mRNA sequences of exons with the wild type nucleotides at positions c.2589G>T (863 V) in exon 26, c.3282G>A (1094E), and c.3345A>G (1115A) in exon 31, and c.3660C>T (1220S) in exon 33 were reported as −36.60 kcal/mol, −28.50 kcal/mol, −28.50 kcal/mol, and −19.60 kcal/mol, respectively. Following the introduction of mutations c.2589G>T (863 V), c.3282G>A (1094E), c.3345A>G (1115A), and c.3660C>T (1220S), the MFE values were adjusted to −35.80 kcal/mol, −25.00 kcal/mol, −28.80 kcal/mol, and −19.60 kcal/mol, respectively. Additionally, these genetic changes brought observable modifications in the secondary structure of the *MYBPC1* mRNA, as illustrated in Figure 6a–c.

## 4 Discussion

The *MYBPC1* gene has been identified as a candidate functional gene associated with carcass traits and meat quality in cattle, pig, and chicken (Verardo et al. 2013; Tong et al. 2014; Li, Cheng, et al. 2020; Li, Pan, et al. 2020). Nevertheless, studies on the association between the expression levels and genetic variants of *MYBPC1* and FA in sheep are scarce. The SCFA are pivotal for maintaining gut and metabolic health (Vinolo et al. 2011). Medium‐chain fatty acids can function as a source of energy and also regulate glucose and lipid metabolism (Ooyama et al. 2009). LCFA have a key function in the growth, development, and health of mammals (Zhang et al. 2019). PUFA can affect the immune function of mammals, when the ratio of n‐6 PUFA to n‐3 PUFA is reduced, the immune response can be enhanced (Karagodina et al. 1993). Intake of n‐3 PUFA can improve vascular and cardiac hemodynamics, triglyceride levels, and enhances autonomic control, while potentially reducing inflammation, thrombosis, and arrhythmias in human (Mozaffarian and Wu 2011). The expression analysis results of this study indicated that the *MYBPC1* expression levels show a positive correlation trend with SCFA and are significantly positively correlated with C4:0, C10:0, and C21:0. Conversely, the *MYBPC1* expression levels are significantly negatively correlated with C18:3n3, MUFA/SFA, and UFA/SFA. Therefore, the low expression levels of *MYBPC1* are more desirable for a beneficial FA composition. Currently, the studies have shown that the main FAs in lamb include C18:0, C16:0, C18:1n9, C18:2n6, C18:3n3, and C20:4n6 (Kamel et al. 2018; Chikwanha et al. 2018). It is well established that SFA is a strong risk factor that can lead to cardiovascular disease by increasing low‐density lipoprotein cholesterol (Mensink 2017). Research studies have established that more beneficial approach to cardiovascular health is to replace dietary SFA with UFA (MUFA and PUFA) (Briggs et al. 2017). Given the significant impact of the composition of FA in lamb on human health, it has received extensive attention in research (Raes et al. 2004). Additionally, the desirable sensory characteristics of meat are closely associated with PUFA and MUFA (Wood et al. 2008). The composition and content of FA in lamb are mainly influenced by genetic, feeding management, and nutritional factors (Mao and Liu 2010). Among them, genetic regulation has become one of the important means of improving meat quality at present. Therefore, based on expression analysis results, the *MYBPC1* gene can serve as an important candidate gene for beneficial FA composition and the quality grade of sheep breeding.

The silent c.2589G>T (863 V), c.3282G>A (1094E), c.3345A>G (1115A), and c.3660C>T (1220S) mutations are the novel variants in the *MYBPC1* gene to exhibit a confirmed association with the FA in Sonid sheep. Even though synonymous mutations preserve the amino acid sequence, they can affect mRNA expression, splicing, stability (Duan et al. 2003; Pagani et al. 2005; Kimchi‐Sarfaty et al. 2007), and secondary structure (Soemedi et al. 2017; Goswami 2018), along with protein translation, folding (Sauna and Kimchi‐Sarfaty 2011), and function (Fung et al. 2014). In this study, the decrease MFE values for the mRNA sequences of exons with the wild type nucleotides at positions c.2589G>T and c.3282G>A. These genetic changes brought observable modifications in the secondary structure of *MYBPC1* mRNA.

In addition, although FAs can be efficiently improved using genotype information (Yokota et al. 2012), there are other factors that contribute to FAs, such as breed, sex, ram (Ponnampalam, Giri, et al. 2014), slaughter age (Rule et al. 1997), and especially in feed (Ponnampalam, Butler, et al. 2014). It is important to note that among the effects of breed (including genetic factors), grassland types (including environment and climate), and grazing habits (including local latitude and culture) on meat FAs of local sheep breeds in a natural grazing system, such as the Mongolian Plateau, the genetic effects including major genes are more efficient for improving the meat quality in local sheep breeds. This will be more practical for consumers who enjoy grazing sheep meat now and in the future. Therefore, the g.170969609A>G, g.170969730C>T, g.170969787C>T, g.171019445C>G, g.171047427G>A, c.3282G>A (1094E), c.3660C>T (1220S), and g.171066159C>G mutations of *MYBPC1* identified might be useful as valuable molecular markers for optimizing the composition and classification of FA in sheep breeding and industry.

In addition, the TF plays a pivotal role in the control of transcription (Levine and Tjian 2003). Upon binding to their corresponding regulatory sequences within the chromatin, the TF initiates a cascade of molecular processes that lead to the recruitment of RNA polymerase and subsequent transcription (Saunders et al. 2006). In a prior study, two pivotal transcription factors, ACSL1 and ASCL2, were identified within the promoter region of the *FAM13A* gene. These TFs' functions primarily focus on the biological processes of adipocyte differentiation, lipid metabolism, and cellular proliferation and apoptosis (Liang et al. 2019). Thus, we used MethPrimer1.0 to predict the methylation of the 2000 bp region in the promoter region (Figure S1a) and found that there was no CpG Island in this region, indicating that the identified SNPs could not change the methylation level of the region. The g.170969609A>G SNP was predicted for the TFs using the Jaspar (the relative profile score threshold set at 80%), the TFs that can be combined with A allele and G allele in the prediction results were deleted, respectively. At the g.170969609A>G SNP, the A allele was changed to G, the motifs of transcription factor LIN54, BARX2, and ZNF341 had disappeared, respectively. Meanwhile, the motifs of transcription factor Cebpa, ZNF354C, HOXA5, and HIC2 had emerged (Figure S1b). Drawing from the findings of association and bioinformatic investigations, we hypothesize that the nucleotide substitution at the g.170969609A>G site might change the motif to affect different TF binding, resulting in up‐or down‐regulation of the promoter activity of *MYBPC1*. Of course, further experiments are still needed to validate this hypothesis. Therefore, our results from the association study may be useful for selecting rams in genetic breeding utilizing markers in the *MYBPC1* gene.

## 5 Conclusions

In conclusion, this study identified six novel mutations of the *MYBPC1* gene associated with the compositions of certain long‐chain FAs, including long‐chain saturated fatty acid, C17:1, n6–polyunsaturated fatty acid, C18:1n9c, and C18:2n6c in Sonid sheep. This study also revealed that the high expression levels of *MYBPC1* were positively correlated with certain saturated fatty acids and negatively correlated with certain unsaturated fatty acid in the LT muscle of Sonid sheep. The results of this study provide a theoretical foundation to improve the meat quality and breeding process of Sonid sheep.

## Author Contributions

**Yilin Wang:** investigation (equal), methodology (equal), validation (equal), writing – original draft (equal). **Xinyue An:** methodology (equal), writing – original draft (equal). **Terigele Li:** methodology (equal). **Takahisa Yamada:** methodology (equal). **Lai Da:** methodology (equal), resources (equal). **Guifang Cao:** methodology (equal), resources (equal). **Ming Cang:** investigation (equal), methodology (equal). **Yongli Song:** funding acquisition (equal), writing – review and editing (equal). **Bin Tong:** conceptualization (equal), project administration (lead), writing – review and editing (lead).

## Ethics Statement

Animal welfare and experimental procedures were carried out in compliance with the guidelines for the Administration of Affairs Concerning Experimental Animals set forth by the Ministry of Science and Technology, China, in 2004. The research protocol received approval from the Institutional Animal Care and Use Ethics Committee at Inner Mongolia University on May 15, 2015, under the authorization number IMU‐2015‐03 for the execution of animal studies.

## Conflicts of Interest

The authors declare no conflicts of interest.

## Supporting information


**Figure S1:** (a) Prediction of CpG methylation at 2000 bp upstream region of the promoter in *MYBPC1*. SNP1: g.170969337C>T, SNP2: g.170969609A>G, SNP3: g.170969682G>A, SNP4: g.170969730C>T, SNP5: g.170969787C>T (b) The g.170969609A>G SNP transcription factor prediction.

**Table S1:** Descriptive statistics for the studied traits in longissimus thoracis with the number of considered Sonid sheep, the mean value and the standard deviation (SD).

**Table S2:** Correlation analyses between any two traits of fatty acid compositions and classes.

**Table S3:** Genotypic frequencies, allelic frequencies, and diversity parameters of 15 mutations in *MYBPC1* of Sonid sheep.

**Table S4:** Linkage disequilibrium as measured by D′ and _*r*_^2^ among 15 mutations in the *MYBPC1*.

**Table S5:** Associations of *MYBPC1* variants with fatty acid composition in longissimus thoracis muscles in Sonid sheep.

## Data Availability

All datasets generated for this study are included in the manuscript.
