# Supplementary material for: Relationships Between Expressions and Variants of the Myosin−Binding Protein C1 Gene and Fatty Acid Composition in Longissimus Thoracis Muscle of Grazing Sonid Sheep
Source: Food Sci Nutr. 2025 Oct 18;13(10):e71057. doi: 10.1002/fsn3.71057 (PMC12535250; doi:10.1002/fsn3.71057)
Supplement: Supplementary file 1 — Figure S1: (a) Prediction of CpG methylation at 2000 bp upstream region of the promoter in MYBPC1. SNP1: g.170969337C>T, SNP2: g.170969609A>G, SNP3: g.170969682G>A, SNP4: g.170969730C>T, SNP5: g.170969787C>T (b) The g.170969609A>G SNP transcription factor prediction. [file FSN3-13-e71057-s006.docx]

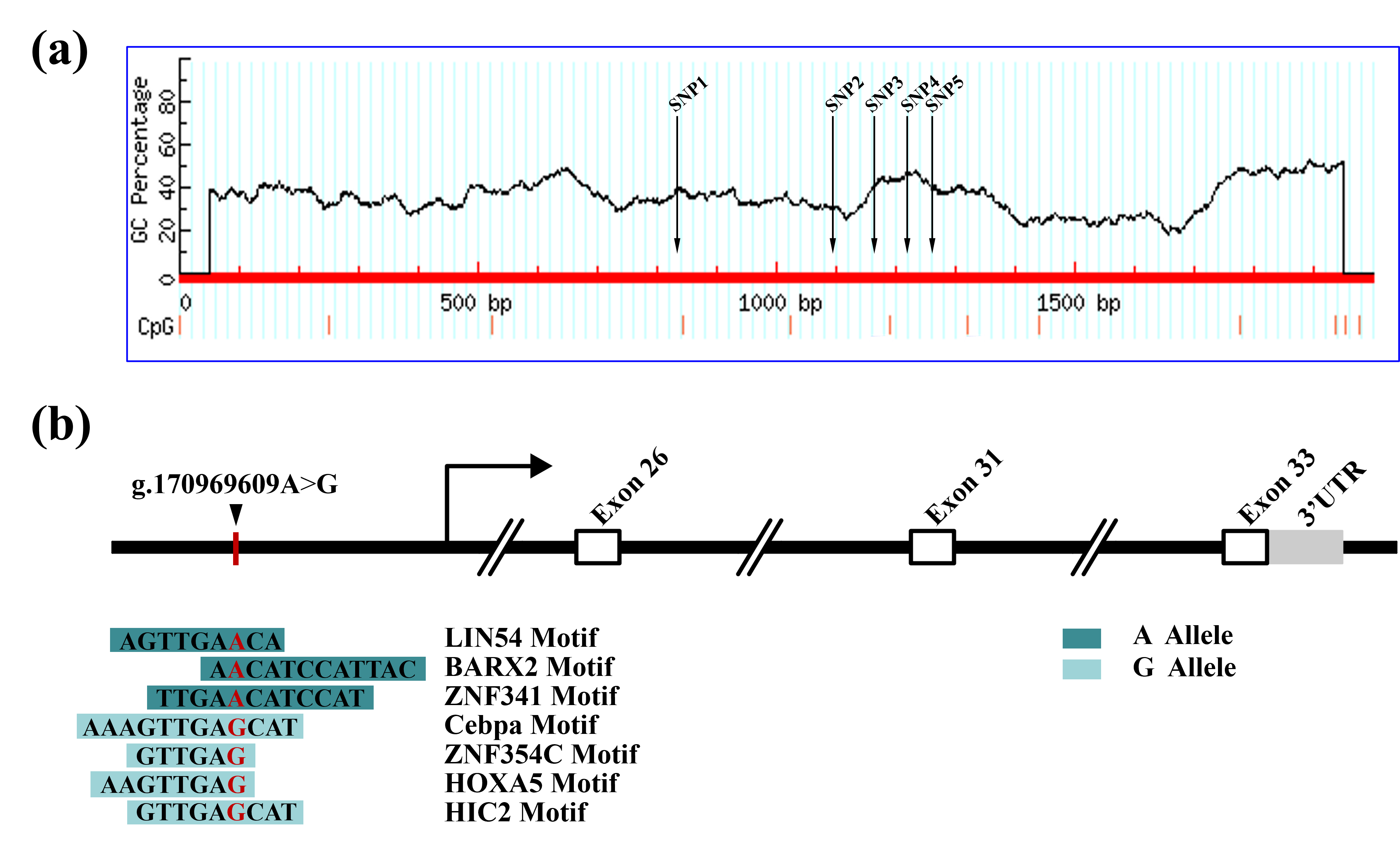


**FIGURE S1** (a) Prediction of CpG methylation at 2000bp upstream region of the promoter in *MYBPC1*. SNP1: g.170969337C>T, SNP2: g.170969609A>G, SNP3: g.170969682G>A, SNP4: g.170969730C>T, SNP5: g.170969787C>T (b) The g.170969609A>G SNP transcription factor prediction.
