# Supplementary material for: Relationships Between Expressions and Variants of the Myosin−Binding Protein C1 Gene and Fatty Acid Composition in Longissimus Thoracis Muscle of Grazing Sonid Sheep
Source: Food Sci Nutr. 2025 Oct 18;13(10):e71057. doi: 10.1002/fsn3.71057 (PMC12535250; doi:10.1002/fsn3.71057)
Supplement: Supplementary file 2 — Table S1: Descriptive statistics for the studied traits in longissimus thoracis with the number of considered Sonid sheep, the mean value and the standard deviation (SD). [file FSN3-13-e71057-s002.doc]

**TABLE S1** Descriptive statistics for the studied traits in longissimus thoracis with the number of considered Sonid sheep, the mean value and the standard deviation (SD)

| **Trait** | **Conctituent** | **N** | **Mean** | **SD** |
| --- | --- | --- | --- | --- |
| Butryic acid | C4:0 | 288 | 1.87 | 0.85 |
| Caproic acid | C6:0 | 288 | 0.94 | 0.59 |
| Capric acid | C10:0 | 288 | 0.32 | 0.12 |
| Undecanoic | C11:0 | 288 | 0.47 | 0.18 |
| Lauric acid | C12:0 | 288 | 0.42 | 0.19 |
| Tridecanoic acid | C13:0 | 288 | 0.67 | 0.40 |
| Myristic acid | C14:0 | 288 | 1.67 | 0.55 |
| Pentadecanoic acid | C15:0 | 288 | 0.79 | 0.39 |
| Palmitic acid | C16:0 | 288 | 16.98 | 3.58 |
| Heptadecanoic acid | C17:0 | 288 | 0.68 | 1.13 |
| Stearic acid | C18:0 | 288 | 9.45 | 1.98 |
| Heneicosylic acid | C21:0 | 288 | 0.63 | 0.62 |
| Behenic acid | C22:0 | 288 | 0.66 | 0.18 |
| Tricosanoic acid | C23:0 | 288 | 0.55 | 0.17 |
| Tetracosanoic acid | C24:0 | 288 | 0.52 | 0.22 |
| Saturated fatty acid | SFA | 288 | 36.64 | 5.60 |
| Myristoleic acid | C14:1 | 288 | 0.63 | 0.32 |
| Palmitoleic acid | C16:1 | 288 | 0.94 | 1.28 |
| Ginkgolic acid | C17:1 | 288 | 0.73 | 0.30 |
| Elaidic acid | C18:1n9t | 288 | 1.71 | 1.53 |
| Oleic acid | C18:1n9c | 288 | 15.81 | 4.39 |
| cis-11-Eicosenopc | C20:1n9 | 288 | 0.73 | 0.19 |
| Erucic acid | C22:1n9 | 288 | 0.60 | 0.26 |
| Monounsaturated fatty acid | MUFA | 288 | 21.16 | 4.91 |
| Linoleic acid | C18:2n6c | 288 | 4.51 | 1.21 |
| α-Linolenic acid | C18:3n3 | 288 | 1.70 | 0.63 |
| dihomo-γ-linolenic acid | C20:3n6 | 288 | 0.46 | 0.15 |
| Arachidonic acid | C20:4n6 | 288 | 0.54 | 0.38 |
| Cis-5,8,11,14,17-Eicosapentaenoic acid | C20:5n3 | 288 | 0.56 | 0.25 |
| Docosahexaaonic acid | C22:6n3 | 288 | 0.47 | 0.13 |
| Polyunsaturated fatty acid | PUFA | 288 | 8.23 | 2.29 |
| Unsaturated fatty acid | UFA | 288 | 29.39 | 5.98 |
| MUFA/SFA | MUFA/SFA | 288 | 0.58 | 0.12 |
| PUFA/SFA | PUFA/SFA | 288 | 0.22 | 0.07 |
| UFA/SFA | UFA/SFA | 288 | 0.80 | 0.14 |
| Short chain fatty acid | SCFA | 288 | 1.87 | 0.85 |
| Medium chain fatty acid | MCFA | 288 | 0.83 | 0.52 |
| Long chain fatty acid | LCFA | 288 | 53.54 | 10.74 |
| Omega 6 Polyunsaturated fatty acids | n-6 | 288 | 5.51 | 1.50 |
| Omega 3 Polyunsaturated fatty acids | n-3 | 288 | 2.72 | 0.81 |
| n-6/n-3 | n-6/n-3 | 288 | 2.02 | 2.28 |
| Essential fatty acid | EFA | 288 | 8.23 | 2.29 |

*Note*: N: number of samples. Mean: mean value. SD: standard deviation.
