# Supplementary material for: Relationships Between Expressions and Variants of the Myosin−Binding Protein C1 Gene and Fatty Acid Composition in Longissimus Thoracis Muscle of Grazing Sonid Sheep
Source: Food Sci Nutr. 2025 Oct 18;13(10):e71057. doi: 10.1002/fsn3.71057 (PMC12535250; doi:10.1002/fsn3.71057)
Supplement: Supplementary file 3 — Table S2: Correlation analyses between any two traits of fatty acid compositions and classes. [file FSN3-13-e71057-s004.doc]

**TABLE S2** Correlation analyses between any two traits of fatty acid compositions and classes

| **Fatty acid** | **C4:0** | **C6:0** | **C10:0** | **C11:0** | **C12:0** | **C13:0** | **C14:0** | **C15:0** | **C16:0** | **C17:0** | **C18:0** | **C21:0** | **C22:0** | **C23:0** |
| --- | --- | --- | --- | --- | --- | --- | --- | --- | --- | --- | --- | --- | --- | --- |
| C4:0 | 1.000 | 0.655** | 0.450** | 0.220 | 0.149 | 0.345** | -0.242* | 0.194 | -0.342** | -0.014 | -0.114 | 0.111 | 0.054 | -0.251 |
| C6:0 | 0.655** | 1.000 | 0.654** | 0.198 | 0.050 | 0.456** | -0.280 | 0.443** | -0.485** | 0.513* | 0.042 | 0.027 | -0.233 | 0.000 |
| C10:0 | 0.450** | 0.654** | 1.000 | 0.536** | 0.579** | 0.605** | -0.034 | 0.430** | -0.324** | -0.132 | -0.423** | -0.189 | 0.150 | -0.290* |
| C11:0 | 0.220 | 0.198 | 0.536** | 1.000 | 0.234* | 0.448** | -0.446** | 0.342** | -0.662** | -0.156 | -0.522** | -0.222 | 0.067 | -0.539** |
| C12:0 | 0.149 | 0.050 | 0.579** | 0.234* | 1.000 | 0.277* | 0.345** | 0.085 | 0.137 | -0.054 | -0.176 | 0.464** | 0.283 | -0.165 |
| C13:0 | 0.345** | 0.456** | 0.605** | 0.448** | 0.277* | 1.000 | -0.347** | 0.249* | -0.282* | 0.002 | -0.107 | 0.038 | 0.073 | 0.015 |
| C14:0 | -0.242* | -0.280 | -0.034 | -0.446** | 0.345** | -0.347** | 1.000 | -0.218** | 0.557** | 0.131 | 0.509** | 0.053 | -0.239 | 0.448** |
| C15:0 | 0.194 | 0.443** | 0.430** | 0.342** | 0.085 | 0.249* | -0.218** | 1.000 | -0.335** | -0.034 | -0.291** | -0.039 | 0.227 | -0.429** |
| C16:0 | -0.342** | -0.485** | -0.324** | -0.662** | 0.137 | -0.282* | 0.557** | -0.335** | 1.000 | 0.104 | 0.621** | 0.006 | -0.070 | 0.655** |
| C17:0 | -0.014 | 0.513* | -0.132 | -0.156 | -0.054 | 0.002 | 0.131 | -0.034 | 0.104 | 1.000 | 0.473** | -0.084 | -0.155 | 0.221 |
| C18:0 | -0.114 | 0.042 | -0.423** | -0.522** | -0.176 | -0.107 | 0.509** | -0.291** | 0.621** | 0.473** | 1.000 | 0.042 | -0.032 | 0.605** |
| C21:0 | 0.111 | 0.027 | -0.189 | -0.222 | 0.464** | 0.038 | 0.053 | -0.039 | 0.006 | -0.084 | 0.042 | 1.000 | 0.440 | -0.161 |
| C22:0 | 0.054 | -0.233 | 0.150 | 0.067 | 0.283 | 0.073 | -0.239 | 0.227 | -0.070 | -0.155 | -0.032 | 0.440 | 1.000 | 0.387 |
| C23:0 | -0.251 | 0.000 | -0.290* | -0.539** | -0.165 | 0.015 | 0.448** | -0.429** | 0.655** | 0.221 | 0.605** | -0.161 | 0.387 | 1.000 |
| C24:0 | -0.240 | -0.810 | -0.086 | -0.108 | -0.060 | -0.141 | 0.195* | -0.186* | 0.234** | 0.120 | 0.256** | -0.055 | 0.116 | 0.391** |
| SFA | -0.089 | -0.067 | -0.269** | -0.689** | 0.061 | -0.066 | 0.562** | -0.246** | 0.912** | 0.181 | 0.706** | 0.154 | -0.071 | 0.678** |
| C14:1 | 0.284* | 0.233 | -0.060 | 0.015 | -0.031 | 0.338* | 0.011 | -0.019 | 0.061 | -0.107 | 0.031 | -0.047 | 0.031 | 0.131 |
| C16:1 | -0.265* | -0.135 | -0.113 | -0.085 | -0.011 | -0.180 | 0.175* | -0.035 | 0.079 | 0.159 | 0.066 | 0.041 | 0.085 | 0.365** |
| C17:1 | 0.283* | 0.530** | 0.357** | 0.476** | 0.260* | 0.389** | -0.076 | 0.421** | -0.291** | -0.021 | -0.024 | 0.164 | 0.443* | -0.223** |
| C18:1n9t | 0.145 | 0.413 | 0.012 | 0.233* | -0.153 | 0.031 | -0.174 | -0.007 | -0.299** | -0.077 | -0.285** | -0.652* | -0.358 | -0.264* |
| C18:1n9c | -0.519** | -0.492** | -0.450** | -0.648** | -0.192 | -0.309** | 0.521** | -0.372** | 0.743** | 0.089 | 0.682** | -0.139 | 0.056 | 0.613** |
| C20:1n9 | -0.229 | -0.505 | -0.693 | -0.265 | -0.871* | -0.130 | -0.233 | -0.057 | -0.045 | 0.181 | -0.187 | 0.877 | -0.145 | -0.189 |
| C22:1n9 | 0.211 | 0.171 | 0.214 | -0.079 | 0.136 | 0.202 | -0.106 | 0.153 | -0.186 | 0.275 | -0.033 | 0.133 | 0.101 | 0.586 |
| MUFA | -0.400** | -0.363* | -0.308** | -0.601** | -0.151 | -0.131 | 0.431** | -0.312** | 0.626** | 0.086 | 0.513** | -0.132 | 0.022 | 0.487** |
| C18:2n6c | -0.250* | -0.310* | -0.256* | -0.538** | -0.056 | -0.358** | 0.234** | -0.202** | 0.652** | 0.025 | 0.361** | -0.117 | -0.072 | 0.699** |
| C18:3n3 | -0.179 | -0.085 | -0.294** | -0.361** | -0.034 | -0.011 | 0.273** | -0.075 | 0.326** | 0.043 | 0.205** | -0.206 | -0.019 | 0.374** |
| C20:3n6 | 0.071 | -0.145 | -0.250 | -0.132 | -0.113 | -0.153 | -0.183 | -0.054 | 0.186 | 0.051 | -0.007 | -0.390 | 0.119 | 0.162 |
| C20:4n6 | 0.038 | 0.040 | 0.080 | -0.102 | 0.261* | 0.017 | 0.257** | -0.185 | 0.266** | 0.007 | 0.202* | 0.290 | -0.248 | -0.027 |
| C20:5n3 | -0.041 | -0.055 | 0.077 | -0.308* | 0.078 | 0.042 | -0.076 | -0.163 | 0.027 | 0.171 | -0.076 | -0.240 | 0.025 | -0.155 |
| C22:6n3 | -0.055 | -0.901 | -0.432* | -0.403* | -0.208 | 0.334 | 0.376* | -0.383* | 0.426** | -0.014 | 0.226 | -0.217 | -0.111 | 0.698** |
| PUFA | -0.369** | -0.383** | -0.181 | -0.469** | -0.072 | -0.223 | 0.113 | 0.022 | 0.422** | 0.043 | 0.030 | -0.206 | -0.130 | 0.394** |
| UFA | -0.468** | -0.448** | -0.321** | -0.651** | -0.158 | -0.195 | 0.396** | -0.238** | 0.670** | 0.084 | 0.420** | -0.203 | -0.060 | 0.525** |
| MUFA/SFA | -0.389** | -0.340* | -0.145 | -0.184** | -0.232* | -0.111 | 0.059 | -0.176* | 0.021 | -0.036 | 0.019 | -0.218 | 0.044 | -0.028 |
| PUFA/SFA | -0.324** | -0.379** | -0.096 | -0.147* | -0.115 | -0.188 | -0.186** | 0.155* | -0.026 | -0.060 | -0.321** | -0.278* | -0.095 | -0.067 |
| UFA/SFA | -0.460** | -0.439** | -0.166 | -0.212** | -0.256** | -0.182 | -0.042 | -0.071 | -0.001 | -0.057 | -0.149* | -0.289* | -0.037 | -0.051 |
| SCFA | 1.000** | 0.655** | 0.450** | 0.220 | 0.149 | 0.345** | -0.242* | 0.194 | -.342** | -0.014 | -0.114 | 0.111 | 0.054 | -0.251 |
| MCFA | 0.391** | 0.714** | 0.750** | 0.322** | 0.424** | 0.448** | -0.168** | 0.346** | -0.222** | -0.081 | -0.404** | -0.080 | 0.127 | -0.268** |
| LCFA | -0.449** | -0.455** | -0.383** | -0.729** | -0.098 | -0.211 | 0.551** | -0.293** | 0.860** | 0.133 | 0.661** | -0.051 | -0.081 | 0.648** |
| n-6 | -0.284** | -0.328* | -0.181 | -0.450** | -0.001 | -0.282* | 0.185** | -0.160* | 0.562** | 0.027 | 0.199** | -0.079 | -0.018 | 0.624** |
| n-3 | -0.190 | -0.127 | -0.231* | -0.418** | -0.051 | 0.028 | 0.212** | -0.086 | 0.344** | 0.042 | 0.076 | -0.246 | -0.028 | 0.443** |
| n-6/n-3 | -0.033 | -0.033 | -0.035 | -0.042 | 0.144 | -0.084 | -0.069 | -0.065 | 0.128 | -0.021 | 0.066 | 0.264 | 0.154 | 0.013 |
| EFA | -0.369** | -0.383** | -0.181 | -0.469** | -0.072 | -0.223 | 0.113 | 0.022 | 0.422** | 0.043 | 0.030 | -0.206 | -0.130 | 0.394** |

*Note*: **p* < 0.05, ***p* < 0.01.

**TABLE S2** (continued)

| **Fatty acid** | **C24:0** | **SFA** | **C14:1** | **C16:1** | **C17:1** | **C18:1n9t** | **C18:1n9c** | **C20:1n9** | **C22:1n9** | **MUFA** | **C18:2n6c** | **C18:3n3** | **C20:3n6** | **C20:4n6** |
| --- | --- | --- | --- | --- | --- | --- | --- | --- | --- | --- | --- | --- | --- | --- |
| C4:0 | -0.240 | -0.089 | 0.284* | -0.265* | 0.283* | 0.145 | -0.519** | -0.229 | 0.211 | -0.400** | -0.250* | -0.179 | 0.071 | 0.038 |
| C6:0 | -0.810 | -0.067 | 0.233 | -0.135 | 0.530** | 0.413 | -0.492** | -0.505 | 0.171 | -0.363* | -0.310* | -0.085 | -0.145 | 0.040 |
| C10:0 | -0.086 | -0.269** | -0.060 | -0.113 | 0.357** | 0.012 | -0.450** | -0.693 | 0.214 | -0.308** | -0.256* | -0.294** | -0.250 | 0.080 |
| C11:0 | -0.108 | -0.689** | 0.015 | -0.085 | 0.476** | 0.233* | -0.648** | -0.265 | -0.079 | -0.601** | -0.538** | -0.361** | -0.132 | -0.102 |
| C12:0 | -0.060 | 0.061 | -0.031 | -0.011 | 0.260* | -0.153 | -0.192 | -0.871* | 0.136 | -0.151 | -0.056 | -0.034 | -0.113 | 0.261* |
| C13:0 | -0.141 | -0.066 | 0.338* | -0.180 | 0.389** | 0.031 | -0.309** | -0.130 | 0.202 | -0.131 | -0.358** | -0.011 | -0.153 | 0.017 |
| C14:0 | 0.195* | 0.562** | 0.011 | 0.175* | -0.076 | -0.174 | 0.521** | -0.233 | -0.106 | 0.431** | 0.234** | 0.273** | -0.183 | 0.257** |
| C15:0 | -0.186* | -0.246** | -0.019 | -0.035 | 0.421** | -0.007 | -0.372** | -0.057 | 0.153 | -0.312** | -0.202** | -0.075 | -0.054 | -0.185 |
| C16:0 | 0.234** | 0.912** | 0.061 | 0.079 | -0.291** | -0.299** | 0.743** | -0.045 | -0.186 | 0.626** | 0.652** | 0.326** | 0.186 | 0.266** |
| C17:0 | 0.120 | 0.181 | -0.107 | 0.159 | -0.021 | -0.077 | 0.089 | 0.181 | 0.275 | 0.086 | 0.025 | 0.043 | 0.051 | 0.007 |
| C18:0 | 0.256** | 0.706** | 0.031 | 0.066 | -0.024 | -0.285** | 0.682** | -0.187 | -0.033 | 0.513** | 0.361** | 0.205** | -0.007 | 0.202* |
| C21:0 | -0.055 | 0.154 | -0.047 | 0.041 | 0.164 | -0.652* | -0.139 | 0.877 | 0.133 | -0.132 | -0.117 | -0.206 | -0.390 | 0.290 |
| C22:0 | 0.116 | -0.071 | 0.031 | 0.085 | 0.443* | -0.358 | 0.056 | -0.145 | 0.101 | 0.022 | -0.072 | -0.019 | 0.119 | -0.248 |
| C23:0 | 0.391** | 0.678** | 0.131 | 0.365** | -0.223** | -0.264* | 0.613** | -0.189 | 0.586 | 0.487** | 0.699** | 0.374** | 0.162 | -0.027 |
| C24:0 | 1.000 | 0.293** | -0.049 | 0.018 | -0.029 | -0.158 | 0.260** | -0.035 | 0.401 | 0.176* | 0.371** | 0.090 | 0.176 | -0.071 |
| SFA | 0.293** | 1.000 | 0.172 | 0.070 | -0.306** | -0.366** | 0.769** | -0.080 | -0.064 | 0.668** | 0.620** | 0.323** | 0.064 | 0.243** |
| C14:1 | -0.049 | 0.172 | 1.000 | 0.064 | -0.054 | -0.063 | 0.110 | -0.070 | 0.042 | 0.167 | -0.149 | 0.165 | -0.135 | -0.291* |
| C16:1 | 0.018 | 0.070 | 0.064 | 1.000 | -0.175* | -0.053 | 0.184* | -0.257 | -0.083 | 0.414** | 0.040 | 0.021 | -0.159 | 0.139 |
| C17:1 | -0.029 | -0.306** | -0.054 | -0.175* | 1.000 | -0.027 | -0.400** | -0.279 | -0.061 | -0.360** | -0.290** | -0.186* | -0.219 | -0.112 |
| C18:1n9t | -0.158 | -0.366** | -0.063 | -0.053 | -0.027 | 1.000 | -0.209* | 0.286 | 0.167 | 0.069 | -0.192 | -0.029 | 0.018 | -0.080 |
| C18:1n9c | 0.260** | 0.769** | 0.110 | 0.184* | -0.400** | -0.209* | 1.000 | 0.027 | -0.162 | 0.920** | 0.547** | 0.297** | -0.253 | 0.279** |
| C20:1n9 | -0.035 | -0.080 | -0.070 | -0.257 | -0.279 | 0.286 | 0.027 | 1.000 | -0.276 | 0.153 | 0.414** | 0.299 | -0.432 | 0.213 |
| C22:1n9 | 0.401 | -0.064 | 0.042 | -0.083 | -0.061 | 0.167 | -0.162 | -0.276 | 1.000 | 0.027 | -0.198 | -0.132 | 0.220 | 0.051 |
| MUFA | 0.176* | 0.668** | 0.167 | 0.414** | -0.360** | 0.069 | 0.920** | 0.153 | 0.027 | 1.000 | 0.454** | 0.254** | -0.312* | 0.231** |
| C18:2n6c | 0.371** | 0.620** | -0.149 | 0.040 | -0.290** | -0.192 | 0.547** | 0.414** | -0.198 | 0.454** | 1.000 | 0.171* | 0.108 | 0.370** |
| C18:3n3 | 0.090 | 0.323** | 0.165 | 0.021 | -0.186* | -0.029 | 0.297** | 0.299 | -0.132 | 0.254** | 0.171* | 1.000 | -0.017 | 0.062 |
| C20:3n6 | 0.176 | 0.064 | -0.135 | -0.159 | -0.219 | 0.018 | -0.253 | -0.432 | 0.220 | -0.312* | 0.108 | -0.017 | 1.000 | -0.271 |
| C20:4n6 | -0.071 | 0.243** | -0.291* | 0.139 | -0.112 | -0.080 | 0.279** | 0.213 | 0.051 | 0.231** | 0.370** | 0.062 | -0.271 | 1.000 |
| C20:5n3 | -0.216 | -0.063 | -0.003 | -0.073 | -0.243 | -0.302 | -0.071 | 0.449 | 0.155 | -0.014 | 0.034 | 0.171 | 0.111 | 0.004 |
| C22:6n3 | 0.536** | 0.411** | 0.692 | 0.000 | -0.001 | 0.140 | 0.273 | -0.388 | 0.407 | -0.094 | 0.371* | 0.183 | 0.043 | -0.073 |
| PUFA | -0.001 | 0.423** | -0.073 | 0.080 | -0.341** | -0.256** | 0.308** | 0.563** | -0.209 | 0.295** | 0.766** | 0.450** | 0.290 | 0.470** |
| UFA | 0.146 | 0.704** | 0.122 | 0.390** | -0.416** | -0.027 | 0.848** | 0.416** | -0.046 | 0.927** | 0.633** | 0.377** | -0.213 | 0.345** |
| MUFA/SFA | -0.060 | 0.012 | 0.068 | 0.419** | -0.200** | 0.550** | 0.516** | 0.248 | 0.062 | 0.729** | 0.055 | 0.051 | -0.367* | 0.124 |
| PUFA/SFA | -0.205** | -0.075 | -0.178 | 0.041 | -0.197** | 0.000 | -0.077 | 0.624** | -0.196 | -0.028 | 0.450** | 0.340** | 0.235 | 0.386** |
| UFA/SFA | -0.143 | -0.032 | -0.003 | 0.400** | -0.263** | 0.499** | 0.382** | 0.613** | -0.015 | 0.606** | 0.230** | 0.194** | -0.271 | 0.251** |
| SCFA | -0.240 | -0.089 | 0.284* | -0.265* | 0.283* | 0.145 | -0.519** | -0.229 | 0.211 | -0.400** | -0.250* | -0.179 | 0.071 | 0.038 |
| MCFA | -0.088 | -0.049 | 0.176 | -0.104 | 0.048 | -0.024 | -0.280** | -0.116 | 0.002 | -0.175** | -0.138* | -0.107 | -0.406** | -0.138 |
| LCFA | 0.246** | 0.905** | 0.115 | 0.267** | -0.371** | -0.192 | 0.900** | 0.155 | -0.087 | 0.884** | 0.669** | 0.387** | -0.081 | 0.344** |
| n-6 | 0.294** | 0.525** | -0.213 | 0.081 | -0.312** | -0.197 | 0.463** | 0.513** | -0.172 | 0.411** | 0.957** | 0.159* | 0.280 | 0.527** |
| n-3 | 0.068 | 0.348** | 0.159 | 0.035 | -0.286** | -0.130 | 0.256** | 0.305 | -0.070 | 0.248** | 0.221** | 0.905** | 0.010 | 0.153 |
| n-6/n-3 | 0.059 | 0.099 | -0.252* | 0.003 | -0.026 | -0.051 | 0.119 | -0.197 | 0.047 | 0.092 | 0.293** | -0.610** | 0.155 | 0.158 |
| EFA | -0.001 | 0.423** | -0.073 | 0.080 | -0.341** | -0.256** | 0.308** | 0.563** | -0.209 | 0.295** | 0.766** | 0.450** | 0.290 | 0.470** |

*Note*: **p* < 0.05, ***p* < 0.01.

**TABLE S2** (continued)

| **Fatty acid** | **C20:5n3** | **C22:6n3** | **PUFA** | **UFA** | **MUFA/SFA** | **PUFA/SFA** | **UFA/SFA** | **SCFA** | **MCFA** | **LCFA** | **n-6** | **n-3** | **n-6/n-3** | **EFA** |
| --- | --- | --- | --- | --- | --- | --- | --- | --- | --- | --- | --- | --- | --- | --- |
| C4:0 | -0.041 | -0.055 | -0.369** | -0.468** | -0.389** | -0.324** | -0.460** | 1.000** | 0.391** | -0.449** | -0.284** | -0.190 | -0.033 | -0.369** |
| C6:0 | -0.055 | -0.901 | -0.383** | -0.448** | -0.340* | -0.379** | -0.439** | 0.655** | 0.714** | -0.455** | -0.328* | -0.127 | -0.033 | -0.383** |
| C10:0 | 0.077 | -0.432* | -0.181 | -0.321** | -0.145 | -0.096 | -0.166 | 0.450** | 0.750** | -0.383** | -0.181 | -0.231* | -0.035 | -0.181 |
| C11:0 | -0.308* | -0.403* | -0.469** | -0.651** | -0.184** | -0.147* | -0.212** | 0.220 | 0.322** | -0.729** | -0.450** | -0.418** | -0.042 | -0.469** |
| C12:0 | 0.078 | -0.208 | -0.072 | -0.158 | -0.232* | -0.115 | -0.256** | 0.149 | 0.424** | -0.098 | -0.001 | -0.051 | 0.144 | -0.072 |
| C13:0 | 0.042 | 0.334 | -0.223 | -0.195 | -0.111 | -0.188 | -0.182 | 0.345** | 0.448** | -0.211 | -0.282* | 0.028 | -0.084 | -0.223 |
| C14:0 | -0.076 | 0.376* | 0.113 | 0.396** | 0.059 | -0.186** | -0.042 | -0.242* | -0.168** | 0.551** | 0.185** | 0.212** | -0.069 | 0.113 |
| C15:0 | -0.163 | -0.383* | 0.022 | -0.238** | -0.176* | 0.155* | -0.071 | 0.194 | 0.346** | -0.293** | -0.160* | -0.086 | -0.065 | 0.022 |
| C16:0 | 0.027 | 0.426** | 0.422** | 0.670** | 0.021 | -0.026 | -0.001 | -0.342** | -0.222** | 0.860** | 0.562** | 0.344** | 0.128 | 0.422** |
| C17:0 | 0.171 | -0.014 | 0.043 | 0.084 | -0.036 | -0.060 | -0.057 | -0.014 | -0.081 | 0.133 | 0.027 | 0.042 | -0.021 | 0.043 |
| C18:0 | -0.076 | 0.226 | 0.030 | 0.420** | 0.019 | -0.321** | -0.149* | -0.114 | -0.404** | 0.661** | 0.199** | 0.076 | 0.066 | 0.030 |
| C21:0 | -0.240 | -0.217 | -0.206 | -0.203 | -0.218 | -0.278* | -0.289* | 0.111 | -0.080 | -0.051 | -0.079 | -0.246 | 0.264 | -0.206 |
| C22:0 | 0.025 | -0.111 | -0.130 | -0.060 | 0.044 | -0.095 | -0.037 | 0.054 | 0.127 | -0.081 | -0.018 | -0.028 | 0.154 | -0.130 |
| C23:0 | -0.155 | 0.698** | 0.394** | 0.525** | -0.028 | -0.067 | -0.051 | -0.251 | -0.268** | 0.648** | 0.624** | 0.443** | 0.013 | 0.394** |
| C24:0 | -0.216 | 0.536** | -0.001 | 0.146 | -0.060 | -0.205** | -0.143 | -0.240 | -0.088 | 0.246** | 0.294** | 0.068 | 0.059 | -0.001 |
| SFA | -0.063 | 0.411** | 0.423** | 0.704** | 0.012 | -0.075 | -0.032 | -0.089 | -0.049 | 0.905** | 0.525** | 0.348** | 0.099 | 0.423** |
| C14:1 | -0.003 | 0.692 | -0.073 | 0.122 | 0.068 | -0.178 | -0.003 | 0.284* | 0.176 | 0.115 | -0.213 | 0.159 | -0.252* | -0.073 |
| C16:1 | -0.073 | 0.000 | 0.080 | 0.390** | 0.419** | 0.041 | 0.400** | -0.265* | -0.104 | 0.267** | 0.081 | 0.035 | 0.003 | 0.080 |
| C17:1 | -0.243 | -0.001 | -0.341** | -0.416** | -0.200** | -0.197** | -0.263** | 0.283* | 0.048 | -0.371** | -0.312** | -0.286** | -0.026 | -0.341** |
| C18:1n9t | -0.302 | 0.140 | -0.256** | -0.027 | 0.550** | 0.000 | 0.499** | 0.145 | -0.024 | -0.192 | -0.197 | -0.130 | -0.051 | -0.256** |
| C18:1n9c | -0.071 | 0.273 | 0.308** | 0.848** | 0.516** | -0.077 | 0.382** | -0.519** | -0.280** | 0.900** | 0.463** | 0.256** | 0.119 | 0.308** |
| C20:1n9 | 0.449 | -0.388 | 0.563** | 0.416** | 0.248 | 0.624** | 0.613** | -0.229 | -0.116 | 0.155 | 0.513** | 0.305 | -0.197 | 0.563** |
| C22:1n9 | 0.155 | 0.407 | -0.209 | -0.046 | 0.062 | -0.196 | -0.015 | 0.211 | 0.002 | -0.087 | -0.172 | -0.070 | 0.047 | -0.209 |
| MUFA | -0.014 | -0.094 | 0.295** | 0.927** | 0.729** | -0.028 | 0.606** | -0.400** | -0.175** | 0.884** | 0.411** | 0.248** | 0.092 | 0.295** |
| C18:2n6c | 0.034 | 0.371* | 0.766** | 0.633** | 0.055 | 0.450** | 0.230** | -0.250* | -0.138* | 0.669** | 0.957** | 0.221** | 0.293** | 0.766** |
| C18:3n3 | 0.171 | 0.183 | 0.450** | 0.377** | 0.051 | 0.340** | 0.194** | -0.179 | -0.107 | 0.387** | 0.159* | 0.905** | -0.610** | 0.450** |
| C20:3n6 | 0.111 | 0.043 | 0.290 | -0.213 | -0.367* | 0.235 | -0.271 | 0.071 | -0.406** | -0.081 | 0.280 | 0.010 | 0.155 | 0.290 |
| C20:4n6 | 0.004 | -0.073 | 0.470** | 0.345** | 0.124 | 0.386** | 0.251** | 0.038 | -0.138 | 0.344** | 0.527** | 0.153 | 0.158 | 0.470** |
| C20:5n3 | 1.000 | 0.094 | 0.328** | 0.096 | 0.007 | 0.342** | 0.137 | -0.041 | -0.146 | 0.039 | 0.079 | 0.429** | -0.291** | 0.328** |
| C22:6n3 | 0.094 | 1.000 | 0.248 | -0.016 | -0.314 | -0.017 | -0.302 | -0.055 | -0.381* | 0.209 | 0.144 | 0.331* | -0.224 | 0.248 |
| PUFA | 0.328** | 0.248 | 1.000 | 0.623** | 0.030 | 0.858** | 0.428** | -0.369** | 0.066 | 0.532** | 0.859** | 0.595** | 0.030 | 1.000** |
| UFA | 0.096 | -0.016 | 0.623** | 1.000 | 0.607** | 0.305** | 0.667** | -0.468** | -0.116* | 0.926** | 0.633** | 0.426** | 0.085 | 0.623** |
| MUFA/SFA | 0.007 | -0.314 | 0.030 | 0.607** | 1.000 | 0.047 | 0.877** | -0.389** | -0.160** | 0.374** | 0.083 | 0.027 | 0.033 | 0.030 |
| PUFA/SFA | 0.342** | -0.017 | 0.858** | 0.305** | 0.047 | 1.000 | 0.509** | -0.324** | 0.087 | 0.097 | 0.616** | 0.472** | -0.029 | 0.858** |
| UFA/SFA | 0.137 | -0.302 | 0.428** | 0.667** | 0.877** | 0.509** | 1.000 | -0.460** | -0.094 | 0.364** | 0.326** | 0.234** | 0.019 | 0.428** |
| SCFA | -0.041 | -0.055 | -0.369** | -0.468** | -0.389** | -0.324** | -0.460** | 1.000 | 0.391** | -0.449** | -0.284** | -0.190 | -0.033 | -0.369** |
| MCFA | -0.146 | -0.381* | 0.066 | -0.116* | -0.160** | 0.087 | -0.094 | 0.391** | 1.000 | -0.190** | -0.066 | -0.027 | 0.004 | 0.066 |
| LCFA | 0.039 | 0.209 | 0.532** | 0.926** | 0.374** | 0.097 | 0.364** | -0.449** | -0.190** | 1.000 | 0.608** | 0.404** | 0.092 | 0.532** |
| n-6 | 0.079 | 0.144 | 0.859** | 0.633** | 0.083 | 0.616** | 0.326** | -0.284** | -0.066 | 0.608** | 1.000 | 0.267** | 0.327** | 0.859** |
| n-3 | 0.429** | 0.331* | 0.595** | 0.426** | 0.027 | 0.472** | 0.234** | -0.190 | -0.027 | 0.404** | 0.267** | 1.000 | -0.564** | 0.595** |
| n-6/n-3 | -0.291** | -0.224 | 0.030 | 0.085 | 0.033 | -0.029 | 0.019 | -0.033 | 0.004 | 0.092 | 0.327** | -0.564** | 1.000 | 0.030 |
| EFA | 0.328** | 0.248 | 1.000** | 0.623** | 0.030 | 0.858** | 0.428** | -0.369** | 0.066 | 0.532** | 0.859** | 0.595** | 0.030 | 1.000 |

*Note*: **p* < 0.05, ***p* < 0.01.
