# Supplementary material for: Relationships Between Expressions and Variants of the Myosin−Binding Protein C1 Gene and Fatty Acid Composition in Longissimus Thoracis Muscle of Grazing Sonid Sheep
Source: Food Sci Nutr. 2025 Oct 18;13(10):e71057. doi: 10.1002/fsn3.71057 (PMC12535250; doi:10.1002/fsn3.71057)
Supplement: Supplementary file 4 — Table S3: Genotypic frequencies, allelic frequencies, and diversity parameters of 15 mutations in MYBPC1 of Sonid sheep. [file FSN3-13-e71057-s005.doc]

**TABLE S3** Genotypic frequencies, allelic frequencies, and diversity parameters of 15 mutations in *MYBPC1* of Sonid sheep

| **SNPs** | **Genotype frequency** | | | **Allele frequency** | | **Diversity parameter** | | | | |
| --- | --- | --- | --- | --- | --- | --- | --- | --- | --- | --- |
|  |  |  |  |  |  | H_o_ | H_e_ | n_e_ | **PIC** | χ² (HWE^2^) |
| g.170969337C>T | GG | GC | CC | G | C |  |  |  |  |  |
|  | 0.734 | 0.245 | 0.021 | 0.857 | 0.143 | 0.754 | 0.246 | 1.326 | 0.215 | 0.003 |
| g.170969609A>G | AA | AG | GG | A | G |  |  |  |  |  |
|  | 0.490 | 0.434 | 0.077 | 0.706 | 0.294 | 0.585 | 0.415 | 1.709 | 0.329 | 0.580 |
| g.170969682G>A | GG | GA | AA | G | A |  |  |  |  |  |
|  | 0.759 | 0.213 | 0.028 | 0.865 | 0.135 | 0.767 | 0.233 | 1.304 | 0.206 | 2.045 |
| g.170969730C>T | CC | CT | TT | C | T |  |  |  |  |  |
|  | 0.689 | 0.290 | 0.021 | 0.834 | 0.166 | 0.723 | 0.277 | 1.383 | 0.239 | 0.650 |
| g.170969787C>T | CC | CT | TT | C | T |  |  |  |  |  |
|  | 0.689 | 0.290 | 0.021 | 0.834 | 0.166 | 0.723 | 0.277 | 1.383 | 0.239 | 0.650 |
| g.171019445C>G | CC | CG | GG | C | G |  |  |  |  |  |
|  | 0.350 | 0.503 | 0.147 | 0.601 | 0.399 | 0.521 | 0.479 | 1.921 | 0.365 | 0.720 |
| c.2589G>T (863V) | GG | GT | TT | G | T |  |  |  |  |  |
|  | 0.643 | 0.329 | 0.028 | 0.808 | 0.192 | 0.689 | 0.311 | 1.451 | 0.262 | 0.962 |
| g.171047427G>A | GG | GA | AA | G | A |  |  |  |  |  |
|  | 0.605 | 0.353 | 0.042 | 0.781 | 0.219 | 0.658 | 0.342 | 1.519 | 0.284 | 0.330 |
| g.171057982G>A | GG | GA | AA | G | A |  |  |  |  |  |
|  | 0.685 | 0.269 | 0.045 | 0.820 | 0.180 | 0.705 | 0.295 | 1.419 | 0.252 | 2.227 |
| g.171058187C>T | CC | CT | TT | C | T |  |  |  |  |  |
|  | 0.769 | 0.206 | 0.024 | 0.872 | 0.128 | 0.777 | 0.223 | 1.286 | 0.198 | 1.547 |
| c.3282G>A (1094E) | GG | GA | AA | G | A |  |  |  |  |  |
|  | 0.234 | 0.465 | 0.301 | 0.467 | 0.533 | 0.502 | 0.498 | 1.991 | 0.374 | 1.239 |
| c.3345A>G (1115A) | AA | AG | GG | A | G |  |  |  |  |  |
|  | 0.899 | 0.094 | 0.007 | 0.946 | 0.054 | 0.897 | 0.103 | 1.114 | 0.097 | 1.791 |
| g.171061056A>C | AA | AC | CC | A | C |  |  |  |  |  |
|  | 0.902 | 0.091 | 0.007 | 0.948 | 0.052 | 0.901 | 0.099 | 1.110 | 0.094 | 2.084 |
| c.3660C>T (1220S) | CC | CT | TT | C | T |  |  |  |  |  |
|  | 0.217 | 0.472 | 0.311 | 0.453 | 0.547 | 0.504 | 0.496 | 1.982 | 0.373 | 0.644 |
| g.171066159C>G | CC | CG | GG | C | G |  |  |  |  |  |
|  | 0.315 | 0.469 | 0.217 | 0.549 | 0.451 | 0.505 | 0.495 | 1.981 | 0.373 | 0.830 |

Note: Ho: observed heterozygosity, He: expected heterozygosity, ne: effective allele numbers, PIC: polymorphism information content, HWE: Hardy-Weinberg equilibrium. The classification was conducted according to the PIC values (PIC value < 0.25, low polymorphism; 0.25 < PIC value < 0.5, moderate polymorphism; and PIC value > 0.5, high polymorphism). No Hardy-Weinberg departure was detected from the obtained genotype frequencies.
