# Supplementary material for: Relationships Between Expressions and Variants of the Myosin−Binding Protein C1 Gene and Fatty Acid Composition in Longissimus Thoracis Muscle of Grazing Sonid Sheep
Source: Food Sci Nutr. 2025 Oct 18;13(10):e71057. doi: 10.1002/fsn3.71057 (PMC12535250; doi:10.1002/fsn3.71057)
Supplement: Supplementary file 5 — Table S4: Linkage disequilibrium as measured by D′ and r 2 among 15 mutations in the MYBPC1. [file FSN3-13-e71057-s003.doc]

TABLE S4 Linkage disequilibrium as measured by D' and *r*2 among 15 mutations in the *MYBPC1*

| **SNPs** | **g.170969337C>T** | **g.170969609A>G** | **g.170969682G>A** | **g.170969730C>T** | **g.170969787C>T** | **g.171019445C>G** | **c.2589G>T** | **g.171047427G>A** | **g.171057982G>A** | **g.171058187C>T** | **c.3282G>A** | **c.3345A>G** | **g.171061056A>C** | **c.3660C>T** |
| --- | --- | --- | --- | --- | --- | --- | --- | --- | --- | --- | --- | --- | --- | --- |
| g.170969609A>G | D'=1.000/ |  |  |  |  |  |  |  |  |  |  |  |  |  |
|  | *r*^2^=0.070 |  |  |  |  |  |  |  |  |  |  |  |  |  |
| g.170969682G>A | D'=1.000/ | D'=1.000/ |  |  |  |  |  |  |  |  |  |  |  |  |
|  | *r*^2^=0.026 | *r*^2^=0.374 |  |  |  |  |  |  |  |  |  |  |  |  |
| g.170969730C>T | D'=1.000/ | D'=1.000/ | D'=1.000/ |  |  |  |  |  |  |  |  |  |  |  |
|  | *r*^2^=0.033 | *r*^2^=0.083 | *r*^2^=0.031 |  |  |  |  |  |  |  |  |  |  |  |
| g.170969787C>T | D'=1.000/ | D'=1.000/ | D'=1.000/ | D'=1.000/ |  |  |  |  |  |  |  |  |  |  |
|  | *r*^2^=0.033 | *r*^2^=0.083 | *r*^2^=0.031 | *r*^2^=1.000 |  |  |  |  |  |  |  |  |  |  |
| g.171019445C>G | D'=0.420/ | D'=0.165/ | D'=0.538/ | D'=0.478/ | D'=0.478/ |  |  |  |  |  |  |  |  |  |
|  | *r*^2^=0.045 | *r*^2^=0.007 | *r*^2^=0.030 | *r*^2^=0.069 | *r*^2^=0.069 |  |  |  |  |  |  |  |  |  |
| c.2589G>T | D'=0.162/ | D'=0.427/ | D'=0.686/ | D'=0.120/ | D'=0.120/ | D'=0.439/ |  |  |  |  |  |  |  |  |
|  | *r*^2^=0.018 | *r*^2^=0.018 | *r*^2^=0.017 | *r*^2^=0.012 | *r*^2^=0.012 | *r*^2^=0.030 |  |  |  |  |  |  |  |  |
| g.171047427G>A | D'=0.369/ | D'=0.247/ | D'=0.389/ | D'=0.653/ | D'=0.653/ | D'=0.376/ | D'=1.000/ |  |  |  |  |  |  |  |
|  | *r*^2^=0.006 | *r*^2^=0.041 | *r*^2^=0.084 | *r*^2^=0.024 | *r*^2^=0.024 | *r*^2^=0.026 | *r*^2^=0.067 |  |  |  |  |  |  |  |
| g.171057982G>A | D'=1.000/ | D'=0.067/ | D'=0.017/ | D'=0.008/ | D'=0.008/ | D'=0.091/ | D'=0.005/ | D'=0.218/ |  |  |  |  |  |  |
|  | *r*^2^=0.037 | *r*^2^=0.002 | *r*^2^=0.000 | *r*^2^=0.000 | *r*^2^=0.000 | *r*^2^=0.001 | *r*^2^=0.000 | *r*^2^=0.003 |  |  |  |  |  |  |
| g.171058187C>T | D'=1.000/ | D'=0.078/ | D'=0.060/ | D'=0.016/ | D'=0.016/ | D'=0.039/ | D'=0.158/ | D'=0.046/ | D'=1.000/ |  |  |  |  |  |
|  | *r*^2^=0.024 | *r*^2^=0.002 | *r*^2^=0.000 | *r*^2^=0.000 | *r*^2^=0.000 | *r*^2^=0.000 | *r*^2^=0.001 | *r*^2^=0.001 | *r*^2^=0.666 |  |  |  |  |  |
| c.3282G>A | D'=0.289/ | D'=0.197/ | D'=0.256/ | D'=0.007/ | D'=0.007/ | D'=0.017/ | D'=0.113/ | D'=0.248/ | D'=0.343/ | D'=1.000/ |  |  |  |  |
|  | *r*^2^=0.016 | *r*^2^=0.014 | *r*^2^=0.009 | *r*^2^=0.000 | *r*^2^=0.000 | *r*^2^=0.000 | *r*^2^=0.003 | *r*^2^=0.015 | *r*^2^=0.023 | *r*^2^=0.128 |  |  |  |  |
| c.3345A>G | D'=0.005/ | D'=0.077/ | D'=0.085/ | D'=0.023/ | D'=0.023/ | D'=0.115/ | D'=0.081/ | D'=1.000/ | D'=0.956/ | D'=1.000/ | D'=1.000/ |  |  |  |
|  | *r*^2^=0.000 | *r*^2^=0.001 | *r*^2^=0.003 | *r*^2^=0.000 | *r*^2^=0.000 | *r*^2^=0.001 | *r*^2^=0.002 | *r*^2^=0.016 | *r*^2^=0.238 | *r*^2^=0.008 | *r*^2^=0.065 |  |  |  |
| g.171061056A>C | D'=0.020/ | D'=0.059/ | D'=0.061/ | D'=0.001/ | D'=0.001/ | D'=0.128/ | D'=0.100/ | D'=1.000/ | D'=0.954/ | D'=1.000/ | D'=1.000/ | D'=1.000/ |  |  |
|  | *r*^2^=0.000 | *r*^2^=0.000 | *r*^2^=0.001 | *r*^2^=0.000 | *r*^2^=0.000 | *r*^2^=0.001 | *r*^2^=0.002 | *r*^2^=0.015 | *r*^2^=0.229 | *r*^2^=0.008 | *r*^2^=0.063 | *r*^2^=0.966 |  |  |
| c.3660C>T | D'=0.136/ | D'=0.205/ | D'=0.287/ | D'=0.051/ | D'=0.051/ | D'=0.023/ | D'=0.137/ | D'=0.013/ | D'=0.274/ | D'=0.896/ | D'=0.662/ | D'=1.000/ | D'=1.000/ |  |
|  | *r*^2^=0.003 | *r*^2^=0.021 | *r*^2^=0.015 | *r*^2^=0.000 | *r*^2^=0.000 | *r*^2^=0.000 | *r*^2^=0.004 | *r*^2^=0.000 | *r*^2^=0.014 | *r*^2^=0.097 | *r*^2^=0.318 | *r*^2^=0.069 | *r*^2^=0.067 |  |
| g.171066159C>G | D'=0.129/ | D'=0.211/ | D'=0.291/ | D'=0.066/ | D'=0.066/ | D'=0.025/ | D'=0.147/ | D'=0.019/ | D'=0.282/ | D'=0.897/ | D'=0.661/ | D'=1.000/ | D'=1.000/ | D'=1.000/ |
|  | *r*^2^=0.002 | *r*^2^=0.023 | *r*^2^=0.016 | *r*^2^=0.001 | *r*^2^=0.001 | *r*^2^=0.000 | *r*^2^=0.004 | *r*^2^=0.000 | *r*^2^=0.014 | *r*^2^=0.097 | *r*^2^=0.314 | *r*^2^=0.070 | *r*^2^=0.067 | *r*^2^=0.993 |
