# Supplementary material for: Relationships Between Expressions and Variants of the Myosin−Binding Protein C1 Gene and Fatty Acid Composition in Longissimus Thoracis Muscle of Grazing Sonid Sheep
Source: Food Sci Nutr. 2025 Oct 18;13(10):e71057. doi: 10.1002/fsn3.71057 (PMC12535250; doi:10.1002/fsn3.71057)
Supplement: Supplementary file 6 — Table S5: Associations of MYBPC1 variants with fatty acid composition in longissimus thoracis muscles in Sonid sheep. [file FSN3-13-e71057-s001.doc]

TABLE S5 Associations of *MYBPC1* variants with fatty acid composition in longissimus thoracis muscles in Sonid sheep

| **Faty acid composition** | **g.170969337C>T** | | **g.170969682G>A** | | **c.2589G>T** | | | **g.171057982G>A** | | | **g.171058187C>T** | | **c.3345A>G** | | **g.171061056A>C** | |
| --- | --- | --- | --- | --- | --- | --- | --- | --- | --- | --- | --- | --- | --- | --- | --- | --- |
|  | **Genotype** | | **Genotype** | | **Genotype** | | | **Genotype** | | | **Genotype** | | **Genotype** | | **Genotype** | |
|  | **CC (210)** | **CT (70)** | **GG (217)** | **GA (61)** | | **GG (184)** | **GT (94)** | **GG (196)** | **GA (77)** | **AA (13)** | **CC (220)** | **CT (59)** | **AA (257)** | **AG (27)** | **AA (258)** | **AC (26)** |
| C4:0 | 1.91 ± 0.10 | 1.70 ± 0.15 | 1.90 ± 0.11 | 1.80 ± 0.11 | | 1.91 ± 0.12 | 1.80 ± 0.11 | 1.96 ± 0.12 | 1.67 ± 0.11 | 1.94 ± 0.43 | 1.94 ± 0.10 | 1.60 ± 0.12 | 1.91 ± 0.10 | 1.64 ± 0.19 | 1.91 ± 0.10 | 1.64 ± 0.19 |
| C6:0 | 0.99 ± 0.09 | 0.70 ± 0.17 | 0.90 ± 0.09 | 1.12 ± 0.21 | | 0.99 ± 0.10 | 0.85 ± 0.15 | 0.93 ± 0.11 | 0.91 ± 0.18 | 1.07 ± 0.24 | 0.96 ± 0.10 | 0.96 ± 0.19 | 0.92 ± 0.09 | 0.89 ± 0.25 | 0.92 ± 0.09 | 0.89 ± 0.25 |
| C10:0 | 0.33 ± 0.01 | 0.30 ± 0.02 | 0.32 ± 0.01 | 0.32 ± 0.02 | | 0.34 ± 0.02 | 0.30 ± 0.02 | 0.31 ± 0.01 | 0.36 ± 0.02 | 0.29 ± 0.02 | 0.31 ± 0.01 | 0.37 ± 0.03 | 0.32 ± 0.01 | 0.34 ± 0.03 | 0.32 ± 0.01 | 0.32 ± 0.02 |
| C11:0 | 0.47 ± 0.01 | 0.48 ± 0.02 | 0.47 ± 0.01 | 0.47 ± 0.02 | | 0.48 ± 0.01 | 0.45 ± 0.02 | 0.46 ± 0.01 | 0.50 ± 0.02 | 0.37 ± 0.05 | 0.46 ± 0.01 | 0.49 ± 0.03 | 0.47 ± 0.01 | 0.49 ± 0.02 | 0.47 ± 0.01 | 0.49 ± 0.03 |
| C12:0 | 0.44 ± 0.02 | 0.38 ± 0.03 | 0.43 ± 0.02 | 0.41 ± 0.04 | | 0.45 ± 0.03 | 0.38 ± 0.02 | 0.42 ± 0.02 | 0.45 ± 0.04 | 0.36 ± 0.04 | 0.42 ± 0.02 | 0.45 ± 0.05 | 0.42 ± 0.02 | 0.48 ± 0.03 | 0.42 ± 0.02 | 0.46 ± 0.03 |
| C13:0 | 0.69 ± 0.05 | 0.56 ± 0.06 | 0.65 ± 0.05 | 0.73 ± 0.12 | | 0.69 ± 0.06 | 0.63 ± 0.06 | 0.72 ± 0.06 | 0.58 ± 0.07 | 0.59 ± 0.15 | 0.70 ± 0.06 | 0.60 ± 0.08 | 0.68 ± 0.05 | 0.48 ± 0.10 | 0.68 ± 0.05 | 0.48 ± 0.10 |
| C14:0 | 1.64 ± 0.04 | 1.73 ± 0.06 | 1.69 ± 0.04 | 1.63 ± 0.07 | | 1.66 ± 0.04 | 1.71 ± 0.06 | 1.66 ± 0.04 | 1.71 ± 0.07 | 1.61 ± 0.16 | 1.65 ± 0.04 | 1.73 ± 0.09 | 1.68 ± 0.04 | 1.62 ± 0.11 | 1.68 ± 0.04 | 1.60 ± 0.11 |
| C15:0 | 0.80 ± 0.03 | 0.78 ± 0.05 | 0.77 ± 0.03 | 0.87 ± 0.05 | | 0.80 ± 0.03 | 0.77 ± 0.05 | 0.77 ± 0.03 | 0.88 ± 0.05 | 0.63 ± 0.14 | 0.78 ± 0.03 | 0.87 ± 0.06 | 0.78 ± 0.03 | 0.88 ± 0.10 | 0.78 ± 0.03 | 0.86 ± 0.10 |
| C16:0 | 16.92 ± 0.26 | 17.24 ± 0.36 | 17.03 ± 0.24 | 16.87 ± 0.47 | | 17.07 ± 0.27 | 16.96 ± 0.35 | 16.97 ± 0.26 | 17.08 ± 0.39 | 17.45 ± 0.94 | 16.89 ± 0.24 | 17.26 ± 0.46 | 17.10 ± 0.23 | 16.55 ± 0.47 | 17.09 ± 0.23 | 16.62 ± 0.48 |
| C17:0 | 0.73 ± 0.17 | 0.50 ± 0.04 | 0.70 ± 0.16 | 0.56 ± 0.06 | | 0.55 ± 0.03 | 0.56 ± 0.03 | 0.74 ± 0.19 | 0.54 ± 0.05 | 0.61 ± 0.08 | 0.73 ± 0.18 | 0.55 ± 0.06 | 0.68 ± 0.14 | 0.78 ± 0.13 | 0.68 ± 0.14 | 0.78 ± 0.13 |
| C18:0 | 9.33 ± 0.14 | 9.84 ± 0.21 | 9.49 ± 0.13 | 9.23 ± 0.26 | | 9.47 ± 0.14 | 9.48 ± 0.22 | 9.55 ± 0.15 | 9.27 ± 0.20 | 9.45 ± 0.53 | 9.50 ± 0.14 | 9.29 ± 0.23 | 9.49 ± 0.12 | 9.37 ± 0.37 | 9.50 ± 0.12 | 9.34 ± 0.38 |
| C21:0 | 0.66 ± 0.11 | 0.53 ± 0.09 | 0.65 ± 0.12 | 0.56 ± 0.05 | | 0.68 ± 0.12 | 0.53 ± 0.07 | 0.66 ± 0.13 | 0.58 ± 0.05 | 0.60 ± 0.06 | 0.65 ± 0.11 | 0.59 ± 0.06 | 0.64 ± 0.10 | 0.54 ± 0.06 | 0.64 ± 0.10 | 0.54 ± 0.06 |
| C22:0 | 0.67 ± 0.03 | 0.62 ± 0.05 | 0.66 ± 0.03 | 0.68 ± 0.05 | | 0.70 ± 0.04 | 0.61 ± 0.03 | 0.65 ± 0.03 | 0.65 ± 0.05 | 0.76 ± 0.04 | 0.66 ± 0.03 | 0.67 ± 0.06 | 0.65 ± 0.03 | 0.73 ± 0.05 | 0.65 ± 0.03 | 0.73 ± 0.05 |
| C23:0 | 0.54 ± 0.02 | 0.57 ± 0.02 | 0.55 ± 0.01 | 0.54 ± 0.03 | | 0.55 ± 0.02 | 0.55 ± 0.02 | 0.56 ± 0.02 | 0.54 ± 0.02 | 0.58 ± 0.06 | 0.55 ± 0.01 | 0.55 ± 0.02 | 0.56 ± 0.01 | 0.50 ± 0.04 | 0.56 ± 0.01 | 0.50 ± 0.04 |
| C24:0 | 0.51 ± 0.02 | 0.56 ± 0.02 | 0.53 ± 0.02 | 0.49 ± 0.03 | | 0.51 ± 0.02 | 0.53 ± 0.03 | 0.50 ± 0.01 | 0.56 ± 0.05 | 0.55 ± 0.08 | 0.51 ± 0.01 | 0.56 ± 0.06 | 0.52 ± 0.02 | 0.57 ± 0.07 | 0.52 ± 0.02 | 0.51 ± 0.04 |
| SFA | 31.01 ± 0.39 | 31.18 ± 0.59 | 31.10 ± 0.38 | 30.85 ± 0.69 | | 31.29 ± 0.39 | 30.73 ± 0.59 | 30.99 ± 0.41 | 31.00 ± 0.59 | 32.88 ± 1.12 | 30.83 ± 0.38 | 31.55 ± 0.67 | 31.19 ± 0.35 | 30.20 ± 0.89 | 31.19 ± 0.35 | 30.15 ± 0.92 |
| C14:1 | 0.64 ± 0.04 | 0.59 ± 0.10 | 0.63 ± 0.04 | 0.64 ± 0.09 | | 0.63 ± 0.05 | 0.65 ± 0.06 | 0.60 ± 0.05 | 0.68 ± 0.08 | 0.61 ± 0.11 | 0.60 ± 0.04 | 0.69 ± 0.09 | 0.65 ± 0.04 | 0.52 ± 0.07 | 0.65 ± 0.04 | 0.52 ± 0.07 |
| C16:1 | 0.86 ± 0.02 | 0.85 ± 0.03 | 0.98 ± 0.12 | 0.82 ± 0.04 | | 0.88 ± 0.02 | 1.08 ± 0.27 | 0.99 ± 0.14 | 0.86 ± 0.04 | 0.83 ± 0.09 | 0.97 ± 0.12 | 0.86 ± 0.04 | 0.95 ± 0.10 | 0.84 ± 0.08 | 0.95 ± 0.10 | 0.86 ± 0.08 |
| C17:1 | 0.72 ± 0.02 | 0.74 ± 0.04 | 0.74 ± 0.02 | 0.67 ± 0.04 | | 0.75 ± 0.02 | 0.66 ± 0.04 | 0.73 ± 0.02 | 0.73 ± 0.04 | 0.59 ± 0.08 | 0.74 ± 0.02 | 0.70 ± 0.04 | 0.72 ± 0.02 | 0.80 ± 0.09 | 0.72 ± 0.02 | 0.79 ± 0.09 |
| C18:1n9t | 1.79 ± 0.19 | 1.48 ± 0.19 | 1.70 ± 0.18 | 1.78 ± 0.32 | | 1.48 ± 0.14 | 2.02 ± 0.32 | 1.87 ± 0.20 | 1.25 ± 0.09 | 1.25 ± 0.12 | 1.83 ± 0.19 | 1.27 ± 0.10 | 1.76 ± 0.17 | 1.15 ± 0.09 | 1.75 ± 0.16 | 1.16 ± 0.11 |
| C18:1n9c | 15.66 ± 0.32 | 16.29 ± 0.44 | 15.75 ± 0.30 | 16.04 ± 0.50 | | 16.03 ± 0.31 | 15.51 ± 0.49 | 15.78 ± 0.33 | 15.92 ± 0.45 | 16.57 ± 1.02 | 15.66 ± 0.30 | 16.22 ± 0.52 | 15.94 ± 0.28 | 15.28 ± 0.68 | 15.93 ± 0.28 | 15.34 ± 0.70 |
| C20:1n9 | 0.74 ± 0.03 | 0.72 ± 0.04 | 0.72 ± 0.03 | 0.76 ± 0.05 | | 0.72 ± 0.03 | 0.78 ± 0.05 | 0.71 ± 0.03 | 0.76 ± 0.06 | 0.90 ± 0.08 | 0.72 ± 0.03 | 0.78 ± 0.05 | 0.72 ± 0.03 | 0.84 ± 0.09 | 0.72 ± 0.03 | 0.84 ± 0.09 |
| C22:1n9 | 0.60 ± 0.04 | 0.56 ± 0.07 | 0.61 ± 0.03 | 0.57 ± 0.08 | | 0.63 ± 0.04 | 0.50 ± 0.06 | 0.57 ± 0.04 | 0.64 ± 0.05 | 0.60 ± 0.13 | 0.59 ± 0.04 | 0.61 ± 0.06 | 0.59 ± 0.03 | 0.65 ± 0.09 | 0.59 ± 0.03 | 0.65 ± 0.09 |
| MUFA | 17.77 ± 0.34 | 18.12 ± 0.47 | 17.82 ± 0.35 | 18.32 ± 0.53 | | 18.15 ± 0.32 | 17.54 ± 0.62 | 17.87 ± 0.37 | 17.90 ± 0.49 | 19.25 ± 1.01 | 17.74 ± 0.34 | 18.38 ± 0.57 | 18.02 ± 0.31 | 17.30 ± 0.73 | 18.02 ± 0.31 | 17.32 ± 0.76 |
| C18:2n6c | 4.49 ± 0.08 | 4.63 ± 0.18 | 4.54 ± 0.09 | 4.41 ± 0.14 | | 4.59 ± 0.10 | 4.41 ± 0.11 | 4.47 ± 0.09 | 4.68 ± 0.16 | 4.55 ± 0.18 | 4.47 ± 0.08 | 4.72 ± 0.19 | 4.53 ± 0.08 | 4.53 ± 0.21 | 4.53 ± 0.08 | 4.55 ± 0.22 |
| C18:3n3 | 1.67 ± 0.05 | 1.83 ± 0.09 | 1.71 ± 0.05 | 1.66 ± 0.09 | | 1.68 ± 0.05 | 1.78 ± 0.07 | 1.67 ± 0.05 | 1.76 ± 0.08 | 1.95 ± 0.20 | 1.67 ± 0.05 | 1.78 ± 0.09 | 1.71 ± 0.04 | 1.57 ± 0.15 | 1.71 ± 0.04 | 1.57 ± 0.16 |
| C20:3n6 | 0.47 ± 0.02 | 0.42 ± 0.06 | 0.45 ± 0.02 | 0.52 ± 0.06 | | 0.47 ± 0.03 | 0.45 ± 0.03 | 0.42 ± 0.02 | 0.54 ± 0.06 | 0.52 ± 0.06 | 0.44 ± 0.02 | 0.51 ± 0.05 | 0.45 ± 0.02 | 0.57 ± 0.12 | 0.45 ± 0.02 | 0.57 ± 0.12 |
| C20:4n6 | 0.53 ± 0.04 | 0.52 ± 0.08 | 0.52 ± 0.04 | 0.55 ± 0.07 | | 0.55 ± 0.04 | 0.52 ± 0.06 | 0.58 ± 0.04 | 0.48 ± 0.06 | 0.40 ± 0.10 | 0.57 ± 0.04 | 0.50 ± 0.06 | 0.54 ± 0.04 | 0.54 ± 0.11 | 0.54 ± 0.04 | 0.54 ± 0.11 |
| C20:5n3 | 0.55 ± 0.03 | 0.59 ± 0.06 | 0.56 ± 0.03 | 0.54 ± 0.05 | | 0.52 ± 0.03 | 0.62 ± 0.05 | 0.54 ± 0.03 | 0.57 ± 0.04 | 0.61 ± 0.08 | 0.55 ± 0.03 | 0.56 ± 0.05 | 0.54 ± 0.03 | 0.62 ± 0.06 | 0.54 ± 0.03 | 0.62 ± 0.06 |
| C22:6n3 | 0.48 ± 0.03 | 0.42 ± 0.03 | 0.47 ± 0.03 | 0.47 ± 0.03 | | 0.43 ± 0.03 | 0.52 ± 0.03 | 0.48 ± 0.02 | 0.43 ± 0.04 | 0.45 ± 0.08 | 0.47 ± 0.02 | 0.42 ± 0.05 | 0.47 ± 0.02 | 0.33 ± 0.04 | 0.47 ± 0.02 | 0.33 ± 0.04 |
| PUFA | 6.18 ± 0.15 | 5.84 ± 0.30 | 6.07 ± 0.16 | 6.02 ± 0.25 | | 6.16 ± 0.17 | 6.03 ± 0.24 | 5.96 ± 0.16 | 6.23 ± 0.29 | 7.22 ± 0.33 | 5.99 ± 0.15 | 6.32 ± 0.35 | 6.07 ± 0.15 | 6.19 ± 0.36 | 6.07 ± 0.15 | 6.22 ± 0.38 |
| UFA | 23.92 ± 0.42 | 23.88 ± 0.62 | 23.86 ± 0.42 | 24.24 ± 0.64 | | 24.28 ± 0.42 | 23.5 ± 0.69 | 23.81 ± 0.44 | 24.05 ± 0.67 | 26.47 ± 1.12 | 23.71 ± 0.40 | 24.58 ± 0.80 | 24.04 ± 0.38 | 23.49 ± 0.90 | 24.04 ± 0.38 | 23.54 ± 0.93 |
| MUFA/SFA | 0.57 ± 0.01 | 0.58 ± 0.01 | 0.57 ± 0.01 | 0.59 ± 0.01 | | 0.58 ± 0.01 | 0.57 ± 0.02 | 0.58 ± 0.01 | 0.57 ± 0.01 | 0.58 ± 0.02 | 0.58 ± 0.01 | 0.58 ± 0.01 | 0.58 ± 0.01 | 0.57 ± 0.02 | 0.58 ± 0.01 | 0.57 ± 0.02 |
| PUFA/SFA | 0.20 ± 0.00 | 0.19 ± 0.01 | 0.20 ± 0.00 | 0.20 ± 0.01 | | 0.20 ± 0.00 | 0.20 ± 0.01 | 0.19 ± 0.00 | 0.20 ± 0.01 | 0.22 ± 0.01 | 0.20 ± 0.00 | 0.20 ± 0.01 | 0.20 ± 0.00 | 0.21 ± 0.01 | 0.20 ± 0.00 | 0.21 ± 0.01 |
| UFA/SFA | 0.77 ± 0.01 | 0.76 ± 0.01 | 0.77 ± 0.01 | 0.79 ± 0.02 | | 0.78 ± 0.01 | 0.76 ± 0.02 | 0.77 ± 0.01 | 0.77 ± 0.01 | 0.80 ± 0.02 | 0.77 ± 0.01 | 0.77 ± 0.02 | 0.77 ± 0.01 | 0.78 ± 0.02 | 0.77 ± 0.01 | 0.78 ± 0.02 |
| SCFA | 1.91 ± 0.10 | 1.70 ± 0.15 | 1.90 ± 0.11 | 1.80 ± 0.11 | | 1.91 ± 0.12 | 1.80 ± 0.11 | 1.96 ± 0.12 | 1.67 ± 0.11 | 1.94 ± 0.43 | 1.94 ± 0.10 | 1.60 ± 0.12 | 1.91 ± 0.10 | 1.64 ± 0.19 | 1.91 ± 0.10 | 1.64 ± 0.19 |
| MCFA | 0.87 ± 0.04 | 0.74 ± 0.04 | 0.85 ± 0.04 | 0.80 ± 0.06 | | 0.88 ± 0.04 | 0.74 ± 0.04 | 0.81 ± 0.03 | 0.87 ± 0.07 | 0.99 ± 0.15 | 0.81 ± 0.03 | 0.92 ± 0.09 | 0.83 ± 0.03 | 0.84 ± 0.09 | 0.83 ± 0.03 | 0.81 ± 0.09 |
| LCFA | 53.38 ± 0.75 | 53.93 ± 1.14 | 53.50 ± 0.74 | 53.67 ± 1.23 | | 54.06 ± 0.76 | 52.95 ± 1.17 | 53.44 ± 0.78 | 53.59 ± 1.18 | 57.02 ± 2.36 | 53.15 ± 0.72 | 54.61 ± 1.37 | 53.83 ± 0.68 | 52.17 ± 1.68 | 53.82 ± 0.68 | 52.19 ± 1.74 |
| n-6 | 4.80 ± 0.10 | 4.75 ± 0.22 | 4.81 ± 0.11 | 4.65 ± 0.18 | | 4.85 ± 0.12 | 4.75 ± 0.14 | 4.77 ± 0.10 | 4.83 ± 0.21 | 4.95 ± 0.23 | 4.78 ± 0.10 | 4.85 ± 0.25 | 4.79 ± 0.10 | 4.89 ± 0.27 | 4.79 ± 0.10 | 4.93 ± 0.28 |
| n-3 | 1.93 ± 0.06 | 1.99 ± 0.12 | 1.96 ± 0.06 | 1.87 ± 0.11 | | 1.91 ± 0.06 | 2.03 ± 0.10 | 1.92 ± 0.06 | 1.93 ± 0.11 | 2.46 ± 0.22 | 1.90 ± 0.06 | 2.07 ± 0.13 | 1.97 ± 0.06 | 1.67 ± 0.17 | 1.97 ± 0.06 | 1.68 ± 0.17 |
| n-6/n-3 | 2.96 ± 0.16 | 3.14 ± 0.47 | 2.99 ± 0.18 | 3.05 ± 0.39 | | 3.11 ± 0.20 | 2.80 ± 0.25 | 2.94 ± 0.17 | 3.27 ± 0.37 | 2.24 ± 0.25 | 3.10 ± 0.18 | 2.77 ± 0.34 | 2.87 ± 0.15 | 4.13 ± 0.72 | 2.87 ± 0.15 | 4.19 ± 0.75 |
| EFA | 6.18 ± 0.15 | 5.84 ± 0.30 | 6.07 ± 0.16 | 6.02 ± 0.25 | | 6.16 ± 0.17 | 6.03 ± 0.24 | 5.96 ± 0.16 | 6.23 ± 0.29 | 7.22 ± 0.33 | 5.99 ± 0.15 | 6.32 ± 0.35 | 6.07 ± 0.15 | 6.19 ± 0.36 | 6.07 ± 0.15 | 6.22 ± 0.38 |
